# Describing the experience of livestock producers from Ohio, USA with ticks and associated diseases

**DOI:** 10.1186/s42522-023-00091-4

**Published:** 2023-11-20

**Authors:** Andreas Eleftheriou, Samantha Swisher, Andréia Arruda, Amanda Berrian, Risa Pesapane

**Affiliations:** 1 Department of Veterinary Preventive Medicine, College of Veterinary Medicine, The Ohio State University, 1920 Coffey Rd, Columbus, OH 43210 USA; 2 School of Environment and Natural Resources, College of Food, Agricultural, and Environmental Sciences, The Ohio State University, 2021 Coffey Rd, Columbus, OH 43210 USA

**Keywords:** Behavioral risk, KAP producer survey, Tick biosecurity, Tick bite prevention, Tick-borne pathogens, Tick-borne zoonoses

## Abstract

**Background:**

As tick ranges expand across North America, the risk of tick bites and tick-borne diseases (TBDs, i.e. diseases or syndromes associated with ticks) rises for humans and animals, making prevention critical. Several U.S. studies have examined knowledge, attitudes, and practices regarding ticks and TBDs for various cohorts of people. However, among occupational groups with high exposure risk from ticks and TBDs are livestock producers, of which we know little about. To help address this gap, an electronic questionnaire was distributed to livestock producers in Ohio, U.S, a state with a robust agricultural sector.

**Methods:**

We generated descriptive statistics and conducted a multiple correspondence analysis followed by hierarchical clustering on principal components to identify producers with similar response profiles.

**Results:**

Responses from 57 producers showed that most (52.6%) think the American dog tick (*Dermacentor variabilis*) is found in Ohio but are unsure about other species. Although several TBDs are present in Ohio, most (> 50%) producers were unsure or unaware of their presence. Interestingly, most (54.4%) thought ticks pose major health risks for their livestock but fewer (49.1%) thought the same for humans. Regardless, most producers did employ at least one prevention method for themselves (96.5%) and their animals (82.5%). Cluster analysis (*n* = 48) identified three groups: the largest (*n* = 21) was considered “aware and cautious” consisting primarily of farm owners. The others generally practiced less prevention and consisted primarily of farm employees.

**Conclusions:**

Our findings indicate that producers in Ohio practice prevention for themselves and their livestock, but demonstrate gaps in knowledge (e.g., TBD occurrence) and attitudes (e.g., health risks) that could be addressed via educational formats we found producers preferred (e.g., extension materials), to encourage informed prevention. This is especially important for farm employees that may practice less prevention because of lower awareness. Our study can guide others in regions faced with similar tick and TBD risks to protect occupational and livestock health.

**Supplementary Information:**

The online version contains supplementary material available at 10.1186/s42522-023-00091-4.

## Background

In North America, hard-bodied ticks and tick-borne diseases (TBDs) are increasing in distribution and incidence across human, domestic animal, and wildlife populations [1]. Multiple drivers, such as climate change, land use, human behavior, and abundance of host reservoirs, have been implicated in the expansion of ticks and their associated disease agents [2]. Several ticks of veterinary and medical concern exist in North America including the Lone star tick (*Amblyomma americanum*), Gulf coast tick (*Amblyomma maculatum*), Rocky Mountain wood tick (*Dermacentor andersoni*), brown dog tick, (*Rhipicephalus sanguineus*), blacklegged ticks (*Ixodes scapularis* and *I. pacificus*), and American dog ticks (*Dermacentor variabilis* and *D. similis*), [1, 3]. Each of these species transmits various disease agents, which span viruses (e.g. Heartland virus), bacteria (e.g. *Anaplasma *spp.), and protozoa (e.g. *Babesia .*spp.), thereby exerting a significant impact on animal and public health as well as economic challenges to the agricultural sector [3]. Therefore, broadly understanding how to prevent and control ticks and TBDs (i.e., biosecurity measures) is a key concern for animal, public, and environmental health.

Knowledge, attitudes, and practices (KAP) surveys are popular in the health and social sciences because they are a resource-efficient mechanism that identifies deficiencies in a target population that can be addressed via evidence-based interventions [4]. Consequently, KAP surveys have been widely used in TBD research targeting either the public [5–7] or select high-risk occupational groups, such as forest workers and farmers [8–10]. Understanding knowledge, attitudes, and practices of occupational groups at high exposure risk to ticks and TBDs can guide targeted education for promoting public and animal health through appropriate biosecurity measures.

Livestock producers are one of the occupational groups with a high exposure risk to ticks and TBDs because of frequent outdoor activities in tick habitats [11]. Seroprevalence surveys for TBDs outside of North America portray producers as a high-risk group [12–14]. Importantly, emerging TBDs in the U.S. were first detected in producers, including Heartland [15] and Bourbon [16] viruses. As invasive tick species, such as the Asian longhorned tick (*Haemaphysalis longicornis*), continue spreading to new geographic areas within the U.S. [17], increasing tick and TBD awareness in high-risk groups becomes paramount. Despite producers having a high exposure risk, their knowledge, attitudes, and practices, regarding ticks and TBDs have received little attention in the U.S. This knowledge gap likely hinders education strategies and awareness campaigns for strengthening biosecurity measures towards ticks and TBDs in the agricultural sector. Although existing educational activities may address tick biosecurity, developing materials guided by the target population’s experience with ticks and TBDs can be more relevant and therefore, more likely to resonate with the intended audience.

Our study objective was to evaluate knowledge, attitudes, and practices (KAP) of livestock producers in Ohio, U.S., regarding ticks and tick-borne diseases (TBDs) for identifying gaps in existing biosecurity measures and guiding educational materials via the delivery formats preferred by our target population. Because Ohio has a robust agricultural economy [18] and is presently faced with expanding and invasive ticks of veterinary and medical concern, this study was very timely and can serve as a model for regions experiencing similar tick expansions and invasions. To accomplish our objective, we conducted an electronic KAP survey of livestock producers from across Ohio using a convenience sample. Herein we report on summary statistics that describe the KAP of livestock producers regarding ticks and TBDs, characterize groups of producers with similar response profiles to inform education strategies, and outline preferred mechanisms for tick and TBD education among producers in Ohio.

## Methods

### Survey design and development

An anonymous KAP electronic questionnaire (Additional file 1) was developed in English and was self-administered using an online survey platform (Qualtrics, Provo, UT, USA). The cross-sectional survey included 67 questions, was mobile-friendly, and had a predicted completion time of 10–20 min (based on Qualtrics).

The questionnaire design consisted of six sections (total 66 questions) presented in the following order: 1) farm information (19 questions), 2) knowledge about tick biology, tick identification (pictures were provided), and TBDs (10 questions), 3) attitudes towards health risks for humans and animals (9 questions), 4) tick exposure and prevention practices for humans (8 questions), 5) tick exposure and prevention practices for animals (9 questions), and 6) demographic information (11 questions). The knowledge section did include one question on the preferred methods for learning about ticks and the demographics section was displayed last. Our questionnaire had several question types including single-answer multiple choice (44 questions), multiple-answer multiple choice (2 questions), multiple-answer multiple choice with ranking (1 question), open-ended (6 questions) and mixed (single- or multiple-answer with open-ended components [13 questions]). Because some questions were conditional to answers from previous questions, not all questions were displayed to each participant.

The questionnaire went through internal validation by our research team and external validation by a small focus group of producers representing the beef, dairy and sheep industries. Both groups were given instructions for how to evaluate the questionnaire and we incorporated their feedback before the questionnaire became accessible to participants.

### Participant recruitment and data collection

The eligibility criteria to participate in the survey were to be a livestock producer of any type (e.g., commercial or hobby) that resides in Ohio and is over 18 years of age. Livestock included camelids, cattle, cervids, bison, equids, poultry, small ruminants, swine, and rabbits. Participants who completed the questionnaire were eligible to receive a financial incentive of five U.S. dollars in the form of a gift card to the business of their choosing within five business days of taking the survey. To receive this incentive, participants needed to complete a separate questionnaire, which ensured that their original questionnaire responses remained anonymous.

Survey participants were recruited in-person and electronically through 297 extension educators covering all 88 counties of Ohio, three commodity groups, two county fairs, and two social media platforms, ensuring a widespread reach. Active recruitment of producers took place from September 2021 to January 2022, but the survey remained accessible until July 25^th^, 2022. This was done due to the project’s timeline; it was not driven by sample size. The study was reviewed by the Institutional Review Board at The Ohio State University (protocol #2021E0922) and determined to meet the criteria for exemption under category 2b. A copy of the survey circulated to producers, including disclosures and consent, is provided as Additional file 1 in the supplementary materials.

### Statistical analysis

Before data analysis, entries deemed fraudulent or duplicate by the online platform’s algorithm were discarded. Next, we excluded entries that were not at least 80% completed. Lastly, a question on whether the farm raises livestock was used as a control measure to ensure that we included only participants that were livestock producers (others were excluded). We generated descriptive statistics (median and range for continuous variables; frequency and percentages for categorical variables) for all the questions and performed a cluster analysis for identifying groups of participants with similar response profiles. Analyses were done in RStudio (ver. 2022.07.2, [19]) using R (ver. 4.2.2, [20]).

To identify groups of producers with similar profiles of attitudes and practices, we used multiple correspondence analysis (MCA) followed by hierarchical clustering on principal components (HCPC). Because of missing data, we performed this analysis on a subset of survey responses. To best identify groups with similar responses, we used broad questions as categorical variables. These included: 1) attitudes towards TBD risk to human or animal health (three categories: very common, occurs occasionally, rare), 2) attitudes towards tick risk to the health of humans (two categories: major issues, minor issues), 3) attitudes towards tick risk to the health of animals (three categories: major issues, minor issues, none), 4) frequency of preventative measures for humans (three categories: always, often, sometimes) and 5) number of preventative measures used in animals (two categories: zero or one, two or more). The latter was used because there was no equivalent frequency measure question included for animals in the survey as preventative measures can vary by livestock species.

We performed agglomerative clustering where we used the Ward’s method as the cluster method and Euclidean distance as the dissimilarity metric to identify producer groups. For each cluster, we provided descriptive statistics (as outlined previously) for responses we thought would be useful for informing targeted education, and covered demography, farm information, knowledge, exposure, surveillance and education regarding ticks and TBDs. Statistical significance was set at *α* = 0.05 and packages “factoextra” [21] and “FactoMineR” [22] were used (see Additional file 11 in the supplementary materials for R code).

## Results

### Participant demography

In total, we recorded 1966 responses. We excluded 1909 records based on 1) fraudulent or duplicate responses (*n* = 1884), primarily dictated by Qualtrics software, 2) less than 80% completion (*n* = 22) and 3) if the farm did not produce livestock (*n* = 2). Unexpectedly, hundreds of responses from internet robot (or “bot”) responses were recorded after dissemination of the questionnaire via social media, an ongoing problem for online surveys [23]. However, after systematic exclusion, we were confident that 57 livestock producers completed at least 80% of the survey and we used this dataset for analyses. Because we could not tabulate the number of people that received but did not take the survey, we could not calculate a response rate.

Of those who reported age (*n* = 51, 89.5%), the median was 40 years (22–65). Most identified as male (93.0%), Caucasian (63.1%), and spoke English (89.5%) as their primary language (Table 1). Most producers were farm owners (59.6%) with an annual income between $55,000 and $130,000 (61.4%). Most of the participants resided at their farms (66.7%) with an overall Ohio county coverage of 39.8% (Additional Fig. 1). Of those who reported acres cultivated (*n* = 47, 82.5%), the median was 70 acres (1–5000). Most farms produced crops (84.2%), raised livestock for food or fiber as commercial establishments (64.9%), and had an established relationship with a veterinarian (80.7%). Most (> 50%) producers were small-scale (based on numbers of animals) and raised primarily beef cattle (63.2%), poultry (57.9%), small ruminants (56.1%), dairy cattle (54.4%), or camelids (50.9%) (Additional Table 1).

**Table 1:** Demographic and general farm information of Ohio-based producers (*n* = 57) that participated in a self-administered and anonymous electronic survey regarding ticks and tick-borne diseases

Question	n (%)
How would you describe yourself?	
Man	53 (93.0%)
Woman	1 (1.7%)
No answer	3 (5.3%)
How would you describe yourself?	
Asian or Pacific Islander	3 (5.3%)
Black or African American	8 (14.0%)
Hispanic or Latino	1 (1.8%)
Native American or Alaskan Native	5 (8.8%)
White or Caucasian	36 (63.1%)
No answer	4 (7.0%)
What is your preferred language?	
English	51 (89.5%)
No answer	6 (10.5%)
What is the highest level of education you have completed?	
Associate’s degree	7 (12.3%)
Bachelor’s degree	11 (19.3%)
High school Diploma or equivalent	11 (19.3%)
Less than a high school diploma	2 (3.5%)
Master’s degree	1 (1.8%)
Professional Degree or Doctorate	3 (5.3%)
Technical, trade, or some college	18 (31.5%)
No answer	4 (7.0%)
What is your annual household income?	
Less than $55,000	6 (10.5%)
$55,000-$130,000	35 (61.4%)
More than $130,000	9 (15.8%)
No answer	7 (12.3%)
Do you live on the farm?	
No	14 (24.5%)
Yes	38 (66.7%)
No answer	5 (8.8%)
Which of the following describes your role on the farm?	
Farm employee	18 (31.6%)
Farm owner	34 (59.6%)
Other	1 (1.8%)
No answer	4 (7.0%)
Is your work on the farm your sole employment?	
No, I also have another job	11 (19.3%)
Yes, I work full-time (40 + hours) on the farm	28 (49.1%)
Yes, I work part-time (< 40 h) on the farm	14 (24.6%)
No answer	4 (7.0%)
How many years of experience do you have working on a farm?	
Less than 5 years	16 (28.1%)
5–15 years	25 (43.8%)
More than 15 years	12 (21.1%)
No answer	4 (7.0%)
Which option best describes the primary purpose of animals on the farm?	
All animals on the farm are pets or produce meat/eggs/fiber for household consumption only	3 (5.3%)
The farm is a commercial farm; animals are raised for food or fiber	37 (64.9%)
The farm is a hobby/backyard farm; animals are raised for show, 4H, sale as pets, etc	15 (26.3%)
No answer	2 (3.5%)
Does the farm have an established relationship with a veterinarian?	
No	6 (10.5%)
Yes	46 (80.7%)
No answer	5 (8.8%)
On average, how often does the veterinarian visit the farm?	
> 4 times per year	12 (21.0%)
2–4 times per year	23 (40.4%)
Not sure	2 (3.5%)
Once a year	9 (15.8%)
No answer	11 (19.3%)

**Fig. 1:**
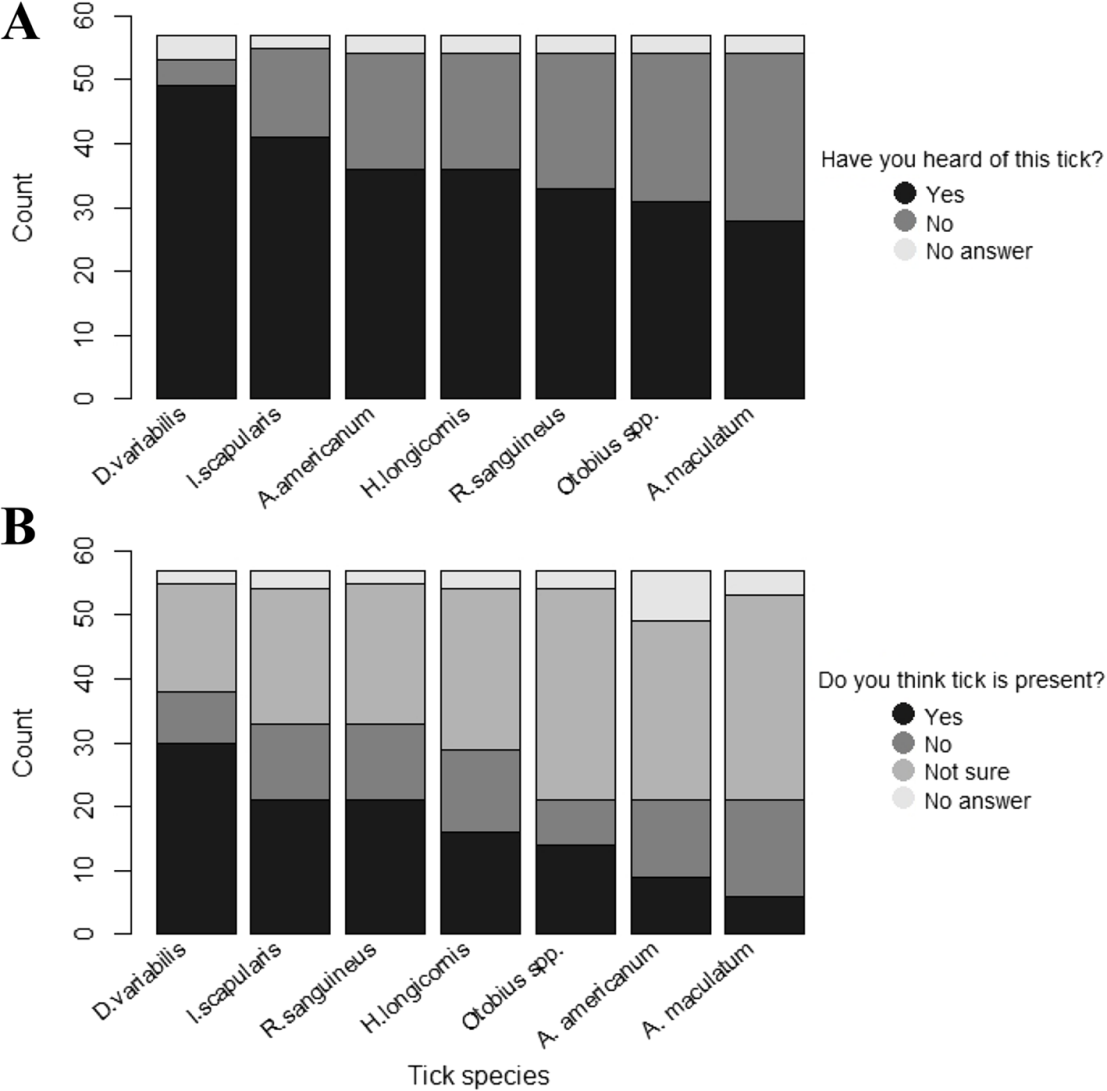
Knowledge of livestock producers (*n* = 57) that participated in an electronic survey regarding tick species relevant to the health of animals and people in Ohio. **A**. Distribution of answers to the question “Have you heard of this species?” **B**. Distribution of answers to the question “Do you think this species is present in Ohio?”. D. variabilis = *Dermacentor variabilis*, I. scapularis = *Ixodes scapularis*, R. sanguineus = *Rhipicephalus sanguineus*, H. longicornis = *Haemaphysalis longicornis*, A. americanum = *Amblyomma americanum*, A. maculatum = *Amblyomma maculatum*. All tick species are present in Ohio. More details in Additional file 5: Table 2

### Knowledge about ticks and TBDs

Although most producers had heard of all six ticks of medical and veterinary concern present in Ohio (Fig. 1a, Additional Table 2), most participants were uncertain of their presence in Ohio, except for the ubiquitous American dog tick (52.6%) (Fig. 1b, Additional Table 2). Most producers were either slightly (38.6%) or moderately confident (41.1%) they could identify a tick and most (64.9%) producers were either slightly or moderately confident identifying whether a tick had fed. Several participants (36.8%) also correctly reported that ticks reach animals by climbing plants and waiting for hosts to walk by. However, another 36.8% reported that ticks get on animals by dropping from trees, and a smaller subset of producers (26.3%) reported that they do so through flying (Table 2).

**Table 2:** Knowledge of Ohio-based producers (*n* = 57) that participated in a self-administered and anonymous electronic survey regarding ticks and tick-borne diseases

Question	n (%)
How confident do you feel identifying ticks?	
Not at all confident: I’m not sure if I could tell a tick from another type of bug	3 (5.3%)
Slightly confident: I can tell that it’s a tick, but am not familiar with individual species	24 (38.6%)
Moderately confident: I know a few common species, but am unsure about the others	22 (42.1%)
Very confident: I can identify most or all of the ticks that I find	6 (10.5%)
No answer	2 (3.5%)
How confident do you feel about determining whether a tick has fed or not?	
Not at all confident	2 (3.5%)
Slightly confident	15 (26.3%)
Moderately confident	22 (38.6%)
Very confident	9 (15.8%)
No answer	9 (15.8%)
How do ticks get onto people or animals?^ab^	
Climb up plants and wait for hosts to walk by	21 (36.8%)
Drop from trees	21 (36.8%)
Fly	15 (26.3%)
Not sure	1 (1.7%)
No answer	12 (21.0%)
Where do you get your information about ticks and tick-borne diseases that affect humans?^a^	
The internet	30 (52.6%)
Extension materials	24 (42.1%)
My doctor	24 (42.1%)
Friends, family, or coworkers	20 (35.1%)
Other	1 (1.8%)
No answer	13 (22.8%)
Where do you get your information about ticks and tick-borne diseases that affect animals?^a^	
Extension materials	29 (50.9%)
My veterinarian	25 (43.9%)
The internet	25 (43.9%)
Producer groups	16 (28.1%)
Friends, family, or coworkers	15 (26.3%)
I don’t get this information from any source	1 (1.8%)
No answer	13 (22.8%)

^a^Sum of percentages may be greater than 100% because participants could choose more than one answer

^b^Ticks get onto animals and people by climbing up grass and waiting for hosts to walk by

**Fig. 2:**
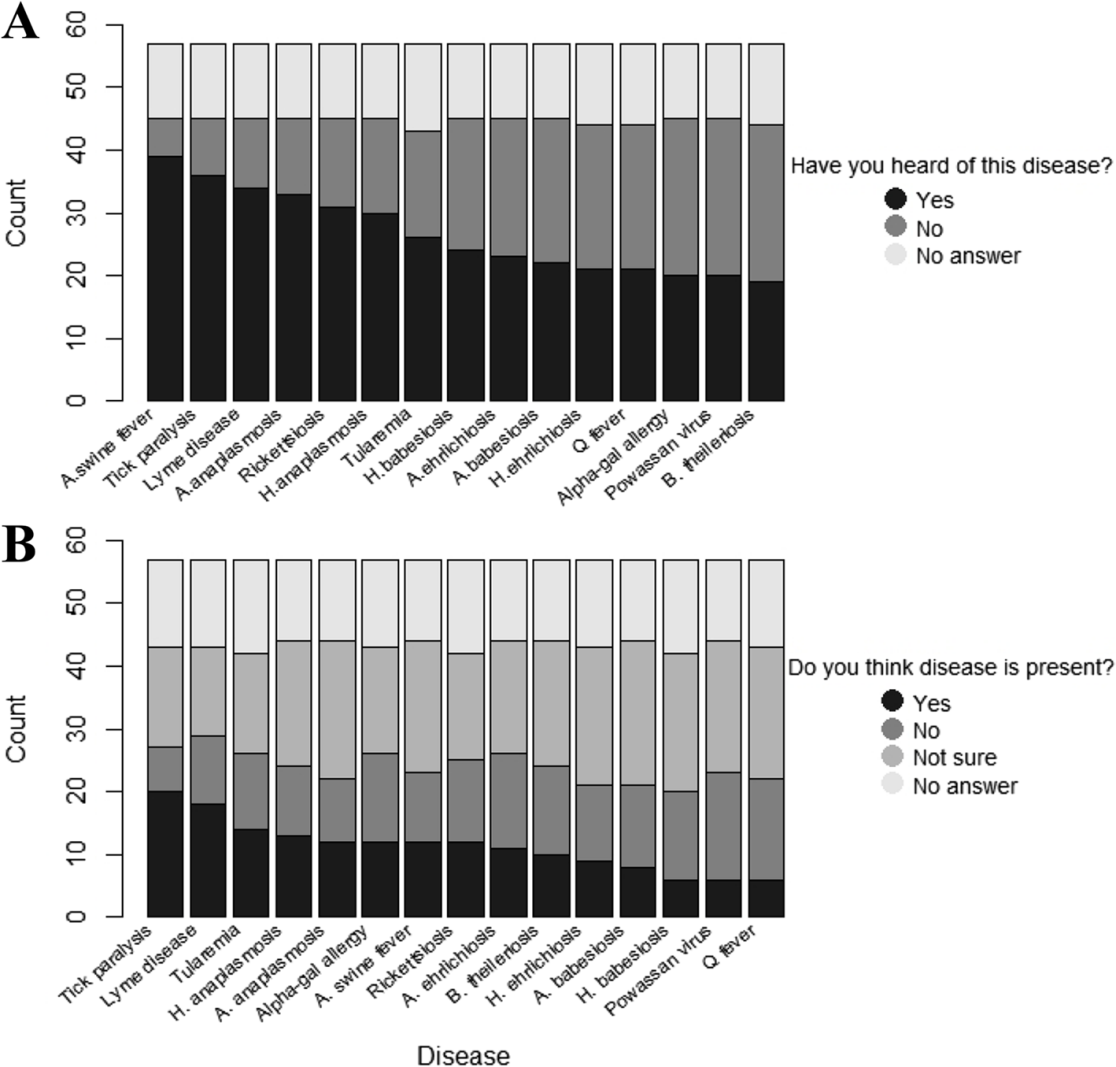
Knowledge of livestock producers (*n* = 57) that participated in an electronic survey regarding tick-borne diseases of animals and people in Ohio. **A**. Distribution of answers to the question “Have you heard of this disease?” **B**. Distribution of answers to the question “Do you think this disease is present?” H. anaplasmosis = human anaplasmosis, A. anaplasmosis = animal anaplasmosis, A. swine fever = African swine fever, A. ehrlichiosis = animal ehrlichiosis, B. theileriosis = bovine theileriosis, H. ehrlichiosis = human ehrlichiosis, A. babesiosis = animal babesiosis, H. babesiosis = human babesiosis. African swine fever and animal babesiosis are not present in Ohio. More details in Additional file 6: Table 3

Most producers had heard of several TBDs such as African swine fever (68.4%), Lyme disease (59.6%) and human anaplasmosis (57.9%). However, fewer had heard of other TBDs, such as bovine theileriosis (43.9%), human ehrlichiosis (40.4%), and animal babesiosis (38.6%). (Fig. 2a, Additional Table 3). Even fewer producers thought TBDs were present in Ohio with tick paralysis (35.1%) and Lyme disease (31.6%) gathering the most support (Fig. 2b, Additional Table 3).

**Table 3:** Attitudes of Ohio-based producers (*n* = 57) that participated in a self-administered and anonymous electronic survey regarding ticks and tick-borne diseases

Question	n (%)
Do you think that ticks pose a risk to your health or the health of your employees/coworkers?	
Yes, major health risks (debilitating or life-threatening)	28 (49.1%)
Yes, minor health risks (short-term or not life-threatening)	27 (47.4%)
No answer	2 (3.5%)
Do you think that ticks pose a risk to the health of the animals that you work with?	
Yes, major health risks (life-threatening or serious loss of production)	31 (54.4%)
Yes, minor health risks (short term illness or minor loss of production)	20 (35.1%)
No	4 (7.0%)
No answer	2 (3.5%)
Considering the past 5 years, have you noticed a change in the number of ticks that you encounter?	
Yes, I used to see more ticks than I do now	14 (24.6%)
Yes, I used to see fewer ticks than I do now	26 (45.6%)
No, the number of ticks I see hasn’t changed	8 (14.0%)
Not sure	6 (10.5%)
No answer	3 (5.3%)
How common do you think tick-borne disease is in humans in Ohio?	
Rare	8 (14.0%)
Occurs occasionally	32 (56.1%)
Very common	13 (22.9%)
No answer	4 (7.0%)
How common do you think tick-borne disease is in livestock in Ohio?	
Rare	6 (10.5%)
Occurs occasionally	28 (49.1%)
Very common	18 (31.6%)
No answer	5 (8.8%)
Do you think that your work on the farm puts you at higher risk for tick-borne disease than the average person in Ohio?	
Yes	46 (80.7%)
No	7 (12.3%)
No answer	4 (7.0%)

There was variability in producer responses regarding information sources for TBDs with most choosing two or more information sources for human (66.7%) and animal TBDs (70.2%). Most popular sources for human TBD information were the internet (52.6%), extension materials (42.1%), and their doctor (42.1%) (Table 2). For animal TBD information, the most popular sources were extension materials (50.9%), their veterinarian (43.9%) and the internet (43.9%) (Table 2).

### Attitudes towards ticks and TBDs

Most producers (80.7%) perceived that their farm puts them at risk of contracting TBDs. While there was similar support for whether ticks pose a major (49.1%) or minor (47.4%) human health risk, this was not the case for animal health, where most producers (54.4%) perceived a major versus a minor risk (35.1%). Importantly, most producers thought TBDs occur occasionally in humans (56.1%) yet fewer thought so for livestock (49.1%) (Table 3). Additionally, most producers reported they noticed a change in the number of tick encounters with 45.6% reporting an increase and 24.6% a decrease when considering the past five years.

### Exposures to ticks and TBDs

Producers perceived handling animals (38.6%), outdoor recreation (19.3%), and crop harvesting (14.0%) as their highest exposure risk, and the majority (50.8%) reported that tick encounters occurred most frequently at work (Table 4). Despite known exposures, most producers had not been diagnosed with a TBD (50.9%, Table 4) and most did not have their livestock diagnosed with a TBD (< 50% responded yes) (Additional Table 4). However, a substantial proportion of producers (~ 40%) did report a suspected or confirmed TBD diagnosis with fewer reporting a TBD diagnosis in livestock (highest was 33.3% diagnosis of anemia). Most producers found 1–5 ticks on themselves (54.5%) within the past year and 1–10 ticks on their livestock (63.2%) at the time of year when ticks are most common (Table 4).

**Table 4:** Exposures of Ohio-based producers (*n* = 57) that participated in a self-administered and anonymous electronic survey regarding ticks and tick-borne diseases

Question	n (%)
What is your highest risk of exposure?	
Crop harvesting	8 (14.0%)
Crop scouting/inspection	3 (5.3%)
Handling animals	22 (38.6%)
Hiking or other outdoor recreation	11 (19.3%)
Hunting	3 (5.3%)
No answer	10 (17.5%)
Approximately how many ticks have you found on yourself in the past year?	
None	13 (22.8%)
1–5	31 (54.4%)
6–10	8 (14.0%)
> 10	3 (5.3%)
No answer	2 (3.5%)
Where do you encounter ticks most frequently?	
At home	15 (26.3%)
At work	29 (50.8%)
During leisure activities (not at home or work)	8 (14.0%)
I don’t encounter ticks	1 (1.8%)
Other:	1 (1.8%)
No answer	3 (5.3%)
Have you ever had a tick-borne disease?	
No: I have not had a tick-borne disease	29 (50.8%)
Yes: My doctor diagnosed me with a specific tick-borne disease	5 (8.8%)
Maybe: I or my doctor suspected a tick-borne disease and started treatment without a specific diagnosis	18 (31.6%)
Prefer not to answer	2 (3.5%)
No answer	3 (5.3%)
At the time of year when ticks are most common in your area, how many ticks do you find on a single animal?	
My livestock don’t get ticks	6 (10.5%)
1–10	36 (63.2%)
11–49	10 (17.5%)
More than 50	2 (3.5%)
No answer	3 (5.3%)

### Practices against ticks and TBDs

Most producers (68.4%) used at least two or more tick preventative measures with the majority (57.9%) reporting they used these often. Prevention most used by producers on themselves included wearing long sleeves and long pants (66.7%), checking for ticks soon after leaving the area (56.1%) and using tick repellents (50.9%) (Table 5). Additionally, 47.9% of producers used at least two or more preventative methods to protect their livestock. The most used preventatives were physical removal (42.1%), insecticidal ear tags (38.6%) and sprays (36.8%) (Table 5). Less than half of the producers (43.9%) had discussed tick prevention with their veterinarian in the past year (Table 5). Although physical removal was the most used prevention, only 14.0% (*n* = 8) correctly stated how to remove ticks from themselves or livestock (i.e., chose only one option: grasp the tick close to its head/mouth and pull straight out).

**Table 5:** Practices of Ohio-based producers (*n* = 57) that participated in a self-administered anonymous electronic survey regarding ticks and tick-borne diseases

Question	n (%)
Which of these personal tick bite prevention strategies do you use when going into areas that you know have a lot of ticks?^a^	
Wear long sleeves and long pants	38 (66.7%)
Checking for ticks soon after leaving the area	32 (56.1%)
Using tick repellent (spray, lotion, or repellent-impregnated clothing)	29 (50.9%)
Shower and changing clothes soon after leaving the area	23 (40.4%)
Other	1 (1.8%)
No answer	2 (3.5%)
How often do you use these strategies when you are in areas that you know have a lot of ticks?	
Always	12 (21.1%)
Often	33 (57.9%)
Sometimes	10 (17.5%)
No answer	2 (3.5%)
Which of the following methods do you use to protect livestock on the farm from ticks?^a^	
Physical removal	24 (42.1%)
Insecticidal ear tag	22 (38.6%)
Spray	21 (36.8%)
Oral/injectable dewormer	20 (35.1%)
Environmental management (e.g. clearing brush)	14 (24.6%)
Pour-on	12 (21.1%)
Dust	9 (15.8%)
We don’t use any tick prevention methods	3 (5.3%)
Dip	2 (3.5%)
No answer	7 (12.3%)
How do you remove ticks from yourself or your animals?^a^	
“Smother” the tick with nail polish, petroleum jelly, alcohol, gasoline, or other substances	24 (42.1%)
Grasp the tick close to its head/mouth using tweezers and pull straight out	21 (36.8%)
Grasp the tick close to its head/mouth and remove using a twisting motion	18 (31.6%)
Burn the tick with a match or lighter	12 (21.1%)
Freeze the tick	4 (7.0%)
Crush the tick before removing	1 (1.8%)
I have never removed a tick	1 (1.8%)
No answer	8 (14.0%)
Has the farm’s veterinarian discussed tick prevention strategies with animal caretakers on the farm?	
No	10 (17.5%)
Yes, but not in the past year	8 (14.0%)
Yes, in the past year	25 (43.9%)
No answer	14 (24.6%)

^a^Sum of percentages may be greater than 100% because participants could choose more than one answer

### Surveillance and education

Approximately 67% of producers had ever submitted a tick for identification. Out of those who had never submitted a tick, the main reason (64.7%) for not doing so was a lack of awareness that this service was available. Most producers would be more likely to submit ticks for identification if they could submit photos (75.4%) versus mailing (33.3%) or submitting through a drop box (28.1%).

Among 57 producers, the most preferred delivery formats for learning about ticks were short video clips online (35.1%, 20 producers ranked #1) and tick identification charts (19.3%, 11 ranked #2). Less preferred formats (received lower rankings) were tick identification phone apps (19.3%), online training modules (21.1%) and in-person training (31.6%).

### Cluster analysis and description

After we excluded records with missing data, 48 producers were included in the cluster analysis. The first and second components of the MCA explained 21.4% and 16.2% of variation in the responses, respectively (Additional Fig. 2a). After retaining the first five principal components to minimize background noise, we found that most of the variables, except the number of animal preventative measures, were significant for cluster selection.

The HCPC generated three producer groups that primarily differed in their responses to frequency of preventative measures in humans and perception of TBD occurrence in humans and livestock (Table 5, Additional Fig. 2b). The major group (*n* = 21), which can be described as “aware and cautious”, considered TBDs in humans to occur occasionally and very commonly (and very commonly in livestock), and used preventative measures against ticks often. The next group (*n* = 15) characterized as “aware yet incautious”, considered TBDs in humans and livestock to occur very commonly (also occasionally in livestock), and practiced prevention sometimes and always (although prevention was deemed less significant). The smallest group (*n* = 12) characterized as “unaware and incautious”, considered TBDs in humans to rarely occur and used preventative measures against ticks sometimes and often.

There were notable differences among the producer clusters regarding their demographic and farm-related responses (Table 6, Additional Table 6). Firstly, the “aware yet incautious” and “unaware and incautious” groups were primarily composed of farm employees. Secondly, the “aware yet incautious” and “aware and cautious” groups mostly composed of producers employed full-time. Thirdly, the “aware yet incautious” group consisted of the most producers with 5–15 years of farm experience. Lastly, the “aware and cautious” group cultivated the highest median number of acres on an average year and reported row crops and prairies/fields as the most common land type on their farm.

**Table 6:** Description of demographic and farm-related variables for Ohio-based livestock producers (*n* = 48) according to their assigned cluster. Number of participants in each cluster are shown and frequency percentages are shown for each variable. This is an abbreviated version. For the complete table, see Additional file 9: Table 6

Cluster	Lives on farm		Role on farm^a^		Sole employment^b^		Experience (years)		Median acres cultivated (range)	Most common land type
1 (*n* = 15) Aware yet incautious	Yes No	73.3 26.7	O E	46.7 53.3	FT PT N	73.3 26.7 0	< 5 5–15 > 15	40.0 53.3 6.7	50^c^ (5–2000)	Lawn or short pasture
2 (*n* = 21) Aware & cautious	Yes No NA	71.4 19.1 9.5	O E NA	76.2 19.0 4.8	FT PT N NA	52.4 23.8 19.0 4.8	< 5 5–15 > 15 NA	19.0 47.6 28.6 4.8	109^d^ (20–5000)	Row crops & prairie/field
3 (*n* = 12) Unaware & incautious	Yes No	58.0 42.0	O E	33.3 66.7	FT PT NA	41.7 33.3 25.0	< 5 5–15 > 15	33.3 50.0 16.7	67.5 (1–200)	Lawn or short pasture

^a^*O* Owner, *E* Employee, *NA* no answer

^b^*FT* Yes, full-time, *PT* Yes, part-time, *N* No, has other job

^c^Calculated on 13 responses

^d^Calculated on 17 responses

We also found interesting differences among producer groups regarding responses related to knowledge, exposure, surveillance, and education (Table 7, Additional Table 7). Firstly, most producers in the “aware yet incautious” group were moderately confident identifying a tick. Secondly, in the “unaware and incautious” group most producers were moderately confident identifying a fed tick. Thirdly, most producers in the “aware yet incautious” and “unaware and incautious” groups perceived highest exposure risk to ticks when handling animals. Lastly, in the “aware yet incautious” group, most producers had discussed tick exposure with a doctor and prevention strategies with a veterinarian in the past year.

**Table 7:** Description of knowledge, exposure, and education variables for Ohio-based livestock producers (*n* = 48) according to their assigned cluster. Number of participants in each cluster are shown and frequency percentages are shown for each variable. This is an abbreviated version. For the complete table, see Additional file 10: Table 7

Cluster	Confident identifying a tick^a^		Confident identifying a fed tick^a^		Highest perceived tick exposure		Place of most frequent tick exposure		Discuss tick exposure at doctor		Discuss tick prevention with veterinarian	
1 (*n* = 15) Aware yet incautious	N S M V	6.6 26.7 66.7 0	N S M V	6.7 40.0 40.0 13.3	Crop-related acts Hunting Handling animals Hiking/outdoors	33.3 0 60.0 6.7	Home Work Leisure None Other	26.7 66.7 6.7 0 0	Yes No Other	80.0 13.3 6.7	Yes, in past year Yes, but not in past year No NA	73.3 13.3 6.7 6.7
2 (*n* = 21) Aware & cautious	N S M V	4.8 47.6 28.6 19.0	N S M V NA^b^	0 23.8 33.3 19.0 23.8	Crop-related acts Hunting Handling animals Hiking/outdoors NA	14.3 9.5 23.8 23.8 28.6	Home Work Leisure None Other	23.8 52.4 23.8 0 0	Yes No Other	38.1 47.6 14.3	Yes, in past year Yes, but not in past year No NA	47.6 9.5 28.6 14.3
3 (*n* = 12) Unaware & incautious	N S M V	8.3 50.0 33.3 8.4	N S M V	8.3 25.0 58.3 8.3	Crop-related acts Hunting Handling animals Hiking/outdoors	8.3 8.3 58.3 25.1	Home Work Leisure None Other	16.7 58.3 8.3 8.3 8.3	Yes No Other	50.0 41.7 8.3	Yes, in past year Yes, but not in past year No NA	33.3 33.3 16.7 16.7

^a^*N* not at all, *S* slightly, *M* moderately, *V* very

^b^*NA* no answer

## Discussion

Our study described the knowledge, attitudes, and practices regarding ticks and tick-borne diseases (TBDs) of livestock producers in Ohio, a neglected occupational group with a high exposure risk in a state with a robust agricultural economy. Our results demonstrate that livestock producers perceived their occupation as placing them at a higher risk of exposure to ticks and TBDs. Producers generally considered ticks and TBDs as important health hazards to themselves and their animals, although they thought TBDs occurred in humans and animals only occasionally. Despite most producers employing multiple tick prevention methods on themselves and their livestock, there was a significant gap in their knowledge regarding ticks and TBDs present in Ohio.

Several ticks of medical and veterinary significance and their associated TBDs are known to occur in Ohio [24]. The American dog tick was thought by most producers to be the only tick species in Ohio, which is not surprising given its widespread distribution [25]. Some species, such as the Asian longhorned tick, may not yet be known to most because they only recently invaded or expanded into Ohio [26]. Although most producers had heard of several TBDs, such as African swine fever, anaplasmosis, human babesiosis, Lyme disease, and spotted fever rickettsiosis (e.g. Rocky Mountain spotted fever), fewer had heard of others, such as alpha-gal syndrome (i.e. red meat allergy), animal babesiosis, bovine theileriosis, human ehrlichiosis, and Q fever. Discrepancies in TBD familiarity are frequent in studies of producers [9] and the U.S. public [27]. However, because of ongoing introduction risks from various sources and the rising importance of underrecognized TBDs, discrepancies should be addressed through targeted messaging that extends beyond local TBDs and includes less common yet significant TBDs.

Despite many TBDs being present in Ohio, most producers were either not sure or unaware they were present. Tick paralysis and Lyme disease were the two TBDs that gathered the most support, albeit less than half of the participants. Although tick paralysis, a form of toxicosis, is associated with various tick species [28], Lyme disease may have been reported as present because its primary vector, the blacklegged tick, is becoming more common in Ohio, and the disease itself receives considerable attention through prevention messaging as it is the most reported vector-borne disease in the country [29]. These results generally agree with other U.S. studies, which showed that the public is familiar with Lyme disease, but not other TBDs, such as Rocky Mountain spotted fever, anaplasmosis, babesiosis, and ehrlichiosis [5, 7]. In our study, it is concerning that 32 producers (56.1%) were unsure or thought animal anaplasmosis was absent given that bovine anaplasmosis, an economically devasting disease, has been detected in at least four beef production farms in Ohio [30]. Perhaps more alarming is that 12 producers (21.1%), seven of which identified as swine producers, reported that African swine fever was present in Ohio, because this is a foreign animal disease that will present a significant economic challenge for the swine industry if it were to invade [31]. To best improve and strengthen biosecurity measures for established, emerging, and transboundary TBDs, clear messaging about ticks and TBDs beyond what is present and common is warranted to educate livestock producers and preserve the food chain.

A large majority of producers reported that their farm puts them at risk of contracting TBDs, with handling animals or their occupation presenting a higher exposure risk for encountering ticks. These findings contrast with other studies of the U.S. public that reported high tick exposure in residential neighborhoods or through outdoor leisure activities [32, 33]. Arguably, because nearly 68% of producers resided at their farm, occupational exposure may be confounded with residential. Nevertheless, working on the farm was explicitly regarded as a high risk for TBDs by our study population. Although most producers reported themselves or their livestock as never diagnosed with TBDs, tick exposure was evident because most reported ticks on themselves and livestock, the latter of which varies in the literature [14, 34]. Although the minority of producers (~ 40%) reported a suspected or confirmed TBD diagnosis, this is higher than farmers from Italy (~ 3%) [8], Hungary (~ 5%) [14], and Bhutan (~ 10%) [35] as well as residents of Connecticut and Maryland, U.S. (~ 28%) [5]. In contrast, we found a lower frequency of TBD diagnosis in livestock (anemia was 33.3%) compared to 45% from a survey study in Norway [36].

Unlike producers from Oklahoma [9], France [10], Norway [36], and Bhutan [35], most producers in Ohio perceived TBDs as a major health hazard to their livestock. However, producers in Ohio were nearly equally divided in whether they perceived TBDs as major or minor hazards to their own health, while other studies contrast these findings [9, 34, 37]. Even so, most producers did not think TBDs were common in people or livestock, which is problematic given the spread and emergence of numerous TBDs in the U.S. [38]. Taken together, producers in Ohio had cautionary attitudes but did not consider TBDs to commonly occur in people or animals.

Our finding that most producers practiced tick prevention for themselves, and their livestock was encouraging. Most producers reported using at least one tick preventative method on themselves with the most common being wearing long-sleeved shirts and long pants, checking for ticks soon after leaving the area, and using tick repellent. These align with previous studies of producers [9, 34] and other exposed groups [5, 6, 39]. Similarly, most producers reported using at least one tick preventative method on their livestock with the most common being physical removal, insecticidal ear tags, and sprays. Studies from other parts of the world also found that some preventative methods are more common than others but across these studies the most common method differed [9, 10, 40]. Nevertheless, according to our findings, extension educators and health professionals, at least in Ohio, could improve on existing tick prevention practices by educating producers on how to correctly remove ticks from themselves or their animals (most reported incorrect methods that may increase TBD transmission risk) and that insecticidal ear tags will not target ticks beyond the head, necessitating alternative prevention, depending on tick species.

Online short videos were most preferred to other modes of learning regarding ticks and TBDs, underscoring the desire of this occupational group to engage with electronically accessible information at their own leisure. Most producers also reported they were more likely to participate in tick surveillance if they were allowed to submit photos, which although useful, would not allow for TBD testing or scrutiny of morphological criteria for differentiation of closely related species. However, because approximately two thirds of producers that never submitted a tick for identification were unaware submitting ticks for surveillance was even a possible service, tick biosecurity in the agricultural sector could benefit from education that includes this information and integrates producers as active stakeholders in surveillance. Although many producers used more than one source for TBD information, the most common sources for human and animal TBDs were the internet and extension materials, respectively. While it is certainly encouraging that most producers had established veterinarian relationships and discussed prevention, our findings suggest that including prevention information regarding animal TBDs in extension materials may reach a wider audience more efficiently. Adding information to extension materials regarding human TBDs could reach the same audience and limit exposure to misinformation via the internet.

Cluster analysis revealed intriguing differences among groups of producers in our study population. Two groups had opposite attitudes and practices, with the largest being “aware and cautious” (i.e. practiced more prevention) and the smallest being “unaware and incautious” (i.e. practiced less prevention). Interestingly, we found that the second largest group was “aware yet incautious”, a pattern previously described [41, 42]. Although relationships between attitudes and practices have been found in other studies [6, 43], in our study the relationship depended on the producer group.

Closer examination of clusters revealed further insight that may be useful for informing education strategies. We found that the “aware and cautious” group consisted primarily of farm owners in contrast to the other two groups, which largely composed of farm employees. Additionally, both “aware” groups were dominated by producers employed full-time. In contrast to the other two groups that perceived handling animals as their highest tick exposure, the “aware and cautious” group consisted primarily of producers that perceived handling animals as only one of their highest exposures (the other being outdoor activities). Interestingly, the same group also cultivated the highest median acres on an average year and their farm primarily consisted of land types most likely to be infested with ticks (e.g., unmowed pastures), which may extend their exposure beyond handling animals. It is noteworthy that most producers in the “aware yet incautious” group had discussed tick exposure with their doctor and prevention with their veterinarian in the past year, possibly indicating that concern and education may increase awareness but not strongly translate to better practices. Jointly, our findings indicate that tick biosecurity may benefit from education that especially targets farm employees and those that do not work full-time at the farm because raising awareness regarding ticks and TBDs may lead to improved practices in these producers.

Despite compelling findings our study does suffer from certain limitations. Firstly, because we had a small convenience sample (57 of at least 30,000 producers) [18], our study is unlikely to have adequately represented the entire producer population in Ohio. Although our sample was biased towards white males, who were willing to engage in a self-administered electronic survey, most producers in Ohio are small-scale [18], which was at least reflected in our survey. Nevertheless, this selection bias may have influenced the results and should be carefully considered. Secondly, as all responses from participants were self-reported, our data may have been susceptible to recall bias. Because many of the questions were broad, this may have had little effect on our results. Future similar studies in Ohio should address these deficiencies through a larger sample size, a probability-based sampling design, and a more targeted selection (e.g. female producers). Aside from these limitations, our study is one of the few in the U.S. to report knowledge, attitudes, and practices, of livestock producers in a state with a robust agricultural economy, providing foundational knowledge to guide messaging for promoting human and animal health within a “One Health” context.

## Conclusions

Our study highlights deficiencies among livestock producers in their knowledge regarding ticks and TBDs, which may have wide public health, economic, and agricultural implications. Yet, it is reassuring that most producers generally had cautionary attitudes towards ticks and TBDs and practiced prevention for themselves and their livestock. Our study provides insightful suggestions for effectively engaging and educating producers to improve their knowledge, attitudes, and practices regarding ticks and TBDs, thereby promoting their health and that of their animals while protecting the food chain.

### Supplementary Information

**Additional file 1.** The electronic questionnaire that was disseminated to Ohio-based livestock producers.

**Additional file 2: Fig. 1.** Number of livestock producers (indicated by color) from each county in Ohio that participated in an electronic survey regarding ticks and tick-borne diseases.

**Additional file 3: Fig. 2.** Cluster analysis of responses from livestock producers (*n* = 48) that participated in an electronic survey regarding ticks and tick-borne diseases (TBDs). A. Factor map demonstrates three clusters (outlined by color) with their respective centers made up of all participants. B. Dendrogram demonstrates the breakdown of participants according to clusters (outlined by color). Variables used for clustering included attitudes towards TBD risk to human or animal health (three categories: very common, occurs occasionally, rare), attitudes towards tick risk to the health of humans (two categories: major issues, minor issues) or animals (three categories: major issues, minor issues, none), and frequency of preventative measures for humans (three categories: always, often, sometimes). The number of preventative measures used in animals (two categories: zero or one, two or more) was deemed insignificant. More details in Additional file 8: Table 5.

**Additional file 4: Table 1.** Description of the livestock species raised on the farms of Ohio-based producers (*n* = 57) that participated in an electronic survey regarding ticks and tick-borne diseases. Number of responses (with percentage) are shown for each question.

**Additional file 5: Table 2.** Knowledge regarding Ohio-relevant tick species of Ohio-based producers (*n* = 57) that participated in an electronic survey regarding ticks and tick-borne diseases (pictures for each tick were included). Number of responses (with percentage) are shown for each question.

**Additional file 6: Table 3.** Knowledge regarding human and animal tick-borne diseases of Ohio-based producers (*n* = 57) that participated in an electronic survey regarding ticks and tick-borne diseases. Number of responses (with percentage) are shown for each question.

**Additional file 7: Table 4.** Livestock exposures to tick-borne diseases (TBDs) reported by Ohio-based producers (*n* = 57) that participated in an anonymous online survey regarding ticks and TBDs.

**Additional file 8: Table 5.** Results from a cluster analysis of responses from Ohio-based livestock producers (*n* = 48) that participated in an electronic survey regarding ticks and tick-borne diseases (TBDs) in Ohio. Variables used for clustering included attitudes towards TBD risk to human or animal health (three categories: very common, occurs occasionally, rare), attitudes towards tick risk to the health of humans (two categories: major issues, minor issues) or animals (three categories: major issues, minor issues, none), frequency of preventative measures for humans (three categories: always, often, sometimes) and number of preventative measures used in animals (two categories: zero or one, two or more). Variable v-test statistics and *p*-values are shown for each cluster that was identified.

**Additional file 9: Table 6.** Description of demographic and farm-related variables for Ohio-based livestock producers (*n* = 48) according to their assigned cluster. Number of participants in each cluster are shown and count percentages are shown for each variable. For more details on clusters see the main text and Additional file 8: Table 5.

**Additional file 10: Table 7.** Description of knowledge, exposure, surveillance, and education variables for Ohio-based livestock producers (*n* = 48) according to their assigned cluster. Number of participants in each cluster are shown and count percentages are shown for each variable. For more details on clusters see the main text and Additional file 8: Table 5.

**Additional file 11.** R code for cluster analysis.

## Data Availability

The dataset used during the current study is available from the corresponding author on reasonable request.
