# Supplementary material for: Describing the experience of livestock producers from Ohio, USA with ticks and associated diseases
Source: One Health Outlook. 2023 Nov 20;5:15. doi: 10.1186/s42522-023-00091-4 (PMC10662443; doi:10.1186/s42522-023-00091-4)
Supplement: Supplementary file 1 — Additional file 1. The electronic questionnaire that was disseminated to Ohio-based livestock producers. [file 42522_2023_91_MOESM1_ESM.docx]

Additional file 1. The electronic questionnaire that was disseminated to Ohio-based livestock producers.

Start of Block: Intro

**This survey is only for livestock producers who reside in Ohio and are over the age of 18. It should take 10-20 minutes to complete.** This survey is part of a study conducted by The Ohio State University that is supported by the USDA. The purpose of this survey is to assess the occupational risk and existing knowledge, attitudes, and practices regarding ticks and tick-borne disease among livestock producers in Ohio. Results of this survey will be used to develop customized training and educational materials about ticks for the livestock production community.
 
PARTICIPATION Your participation in this survey is voluntary. You may refuse to take part in the research or exit the survey at any time without penalty. You are free to decline to answer any particular question you do not wish to answer for any reason.

 INCENTIVE For completing this survey, you are eligible for a monetary compensation of $5.00 in the form of a gift card to the business of your choosing. To claim your compensation, enter your name and email address at the end of the survey. This information is collected separately from your responses and will be used only for issuing compensation. You will receive an email with a private link to your gift card within 5 business days. Please note that this compensation is considered taxable income.

 RISKS There are no foreseeable risks involved in participating in this study other than those encountered in day-to-day life.

 CONFIDENTIALITY Your survey answers will be sent to a link at Qualtrics.com where data will be stored in a password protected electronic format. Qualtrics will not collect your IP address and any identifying information you provide voluntarily for compensation will be stored separately from your answers. Therefore, your responses will remain confidential. Your de-identified responses may be used or shared with other researchers without your additional informed consent.

 CONTACT If you have questions about this survey, please contact the primary investigator, Dr. Risa Pesapane, by email at ticks@osu.edu. For questions about your rights as a participant in this study or to discuss other study-related concerns or complaints with someone who is not part of the research team, you may contact the Office of Responsible Research Practices at 1-800-678-6251 or hsconcerns@osu.edu.

 ELECTRONIC CONSENT: You may save this consent form for your records. **Clicking on the next arrow “-->” button indicates that:**
 • You have read the above information
 • You voluntarily agree to participate
 • You are 18 years of age or older

End of Block: Intro

Start of Block: Farm Information

Q68 For the following questions, if you own or work on multiple farms, please consider the farm where you spend most of your time.

Q20 In what county is the farm where you work located?

Adams Allen Ashland Ashtabula Athens Auglaize Belmont Brown Butler Carroll Champaign Clark Clermont Clinton Columbiana Coshocton Crawford Cuyahoga Darke Defiance Delaware Erie Fairfield Fayette Franklin Fulton Gallia Geauga Greene Guernsey Hamilton Hancock Hardin Harrison Henry Highland Hocking Holmes Huron Jackson Jefferson Knox Lake Lawrence Licking Logan Lorain Lucas Madison Mahoning Marion Medina Meigs Mercer Miami Monroe Montgomery Morgan Morrow Muskingum Noble Ottawa Paulding Perry Pickaway Pike Portage Preble Putnam Richland Ross Sandusky Scioto Seneca Shelby Stark Summit Trumbull Tuscarawas Union Van Wert Vinton Warren Washington Wayne Williams Wood Wyandot

Q21 Does the farm produce crops?

- Yes
- No

Q22 Approximately how many acres are cultivated on an average year?

________________________________________________________________

Q23 Does the farm raise livestock?

- Yes
- No

Q24 Please tell us more about the livestock raised on the farm:

|  | Is this species present on the farm? | | Do these animals spend any time on pasture? | | |
| --- | --- | --- | --- | --- | --- |
|  | Yes | No | Yes | No | N/A |
| Camelids (e.g. llamas, alpacas) |  |  |  |  |  |
| Cattle, beef |  |  |  |  |  |
| Cattle, dairy |  |  |  |  |  |
| Cervids (e.g. deer, elk) or bison |  |  |  |  |  |
| Equids (e.g. horses, donkeys) |  |  |  |  |  |
| Poultry (e.g. chickens, turkeys, ducks) |  |  |  |  |  |
| Small ruminants (e.g. sheep, goats) |  |  |  |  |  |
| Swine |  |  |  |  |  |
| Rabbits |  |  |  |  |  |
| Other: |  |  |  |  |  |

Q25 How many weaned camelids are present on the farm?

- Fewer than 10
- 10 to 50
- More than 50

Q26 How many weaned beef cattle are present on the farm?

- Fewer than 20
- 20 to 100
- More than 100

Q27 How many weaned dairy cows are present on the farm?

- Fewer than 20
- 20 to 200
- More than 200

Q28 How many weaned cervids and/or bison are present on the farm?

- Fewer than 10
- 10 to 50
- More than 50

Q29 How many weaned equids are present on the farm?

- Fewer than 25
- 25 to 50
- More than 50

Q30 How many poultry are present on the farm?

- Fewer than 20,000
- 20,000 to 100,000
- More than 100,000

Q31 How many weaned small ruminants are present on the farm?

- Fewer than 25
- 25 to 100
- More than 100

Q32 How many weaned swine are present on the farm?

- Fewer than 25
- 25 to 1000
- More than 1000

Q26 How many weaned rabbits are present on the farm?

- Fewer than 100
- 100 to 600
- More than 600

Q27 Which option best describes the primary purpose of animals on the farm?

- The farm is a commercial farm; animals are raised for food or fiber.
- The farm is a hobby/backyard farm; animals are raised for show, 4H, sale as pets, etc.
- All animals on the farm are pets or produce meat/eggs/fiber for household consumption only.

Q28 Does the farm have an established relationship with a veterinarian?

- Yes
- No

Q29 On average, how often does the veterinarian visit the farm?

- >4 times per year
- 2-4 times per year
- Once a year
- Never - we always take animals that need care to their office
- Not sure

Q30 How much of the farm is made up of the following types of land?

Row crops : _______

Wood lot : _______

Prairie/field (unmowed or infrequently mowed) : _______

Lawn or short pasture (frequently mowed or heavily grazed) : _______

Other : _______

Total : ________

Q31 How often do you see the following types of wildlife on the farm?

|  | Often | Occasionally | Never |
| --- | --- | --- | --- |
| Deer |  |  |  |
| Coyotes |  |  |  |
| Foxes |  |  |  |
| Opossums |  |  |  |
| Squirrels & chipmunks |  |  |  |
| Mice & voles |  |  |  |

End of Block: Farm Information

Start of Block: Knowledge

Q34 Questions about specific tick species:
(Click on pictures to make them larger)

|  | Have you heard of this species? | | Do you think this species is present in Ohio? | | | Have you ever seen this species on the farm? | | |
| --- | --- | --- | --- | --- | --- | --- | --- | --- |
|  | Yes | No | Yes | No | Not sure | Yes | No | Not sure |
| American Dog Tick (*Dermacentor variabilis*) |  |  |  |  |  |  |  |  |
| Asian Longhorned Tick (*Haemaphysalis longicornis*) |  |  |  |  |  |  |  |  |
| Blacklegged/Deer Tick (*Ixodes scapularis*) |  |  |  |  |  |  |  |  |
| Brown Dog Tick (*Rhipicephalus sanguineus*) |  |  |  |  |  |  |  |  |
| Ear Tick (*Otobius* spp) |  |  |  |  |  |  |  |  |
| Gulf Coast Tick (*Amblyomma maculatum*) |  |  |  |  |  |  |  |  |
| Lone Star Tick (*Amblyomma americanum*) |  |  |  |  |  |  |  |  |

Q33 How confident do you feel about identifying ticks?

- Very confident: I can identify most or all of the ticks that I find
- Moderately confident: I know a few common species, but am unsure about the others
- Slightly confident: I can tell that it's a tick, but am not familiar with individual species
- Not at all confident: I'm not sure if I could tell a tick from another type of bug

Q34 How confident do you feel about determining whether a tick has fed or not?

- Very confident
- Moderately confident
- Slightly confident
- Not at all confident

Q35 Which of the following activities do you think represents the highest risk for exposure to ticks?

- Crop scouting/inspection
- Crop harvesting
- Handling animals
- Hiking or other outdoor recreation
- Hunting
- Other: __________________________________________________

Q36 How do ticks get onto people or animals? (Check all that apply)

- Climb up plants and wait for hosts to walk by
- Drop from trees
- Fly
- Not sure

Q37 Questions about diseases carried by ticks:

|  | Have you heard of this disease? | | Do you think it occurs in Ohio? | | |
| --- | --- | --- | --- | --- | --- |
|  | Yes | No | Yes | No | Not sure |
| African Swine Fever |  |  |  |  |  |
| Alpha Gal Syndrome (aka Red Meat Allergy) |  |  |  |  |  |
| Anaplasmosis, human |  |  |  |  |  |
| Anaplasmosis, animal |  |  |  |  |  |
| Babesiosis, human |  |  |  |  |  |
| Babesiosis, animal (aka piroplasmosis, red water, tick fever) |  |  |  |  |  |
| Borreliosis (aka Lyme Disease) |  |  |  |  |  |
| Bovine Theileriosis |  |  |  |  |  |
| Ehrlichiosis, human |  |  |  |  |  |
| Ehrlichiosis, animal (aka heartwater) |  |  |  |  |  |
| Powassan Virus |  |  |  |  |  |
| Q-Fever (*Coxiella burnetii*) |  |  |  |  |  |
| Spotted Fever Rickettsiosis (e.g. Rocky Mountain Spotted Fever) |  |  |  |  |  |
| Tick Paralysis |  |  |  |  |  |
| Tularemia |  |  |  |  |  |

Q38 Where do you get your information about ticks and tick-borne diseases that **affect humans**? (Check all that apply)

- My doctor
- The internet
- Extension materials
- Friends, family, or coworkers
- Other: __________________________________________________
- I don't get this information from any source

Q39 Where do you get your information about ticks and tick-borne diseases that **affect animals**? (Check all that applly)

- My veterinarian
- The internet
- Extension materials
- Producer groups
- Friends, family, or coworkers
- Other: _________________________________________________
- I don't get this information from any source

Q69 Which of the following ways to learn more about ticks would you find most helpful? Please rank in order of preference.

______ Short video clips online

______ Written materials from extension agent

______ Tick identification chart to hang in the barn

______ Tick ID app for your phone

______ Online training modules

______ In-person training

Q41 What additional information would you be interested in learning about ticks or tick-borne disease?

________________________________________________________________

End of Block: Knowledge

Start of Block: Attitudes

Q42 Do you think that ticks pose a risk to **your health** or the **health of your employees/coworkers**?

- Yes, major health risks (debilitating or life-threatening)
- Yes, minor health risks (short-term or not life-threatening)
- No

Q43 Do you think that ticks pose a risk to the **health of the animals** that you work with?

- Yes, major health risks (life-threatening or serious loss of production)
- Yes, minor health risks (short term illness or minor loss of production)
- No

Q44 What types of ticks are you most concerned about?

________________________________________________________________

Q45 Which tick-borne disease(s) are you most concerned about?

________________________________________________________________

Q46 What time(s) of year do you feel ticks are most commonly seen in your area? [Check all that apply]

- Spring (March-May)
- Summer (June-August)
- Fall (September-November)
- Winter (December - February)

Q47 Considering the past 5 years, have you noticed a change in the number of ticks that you encounter?

- Yes, I used to see more ticks than I do now
- Yes, I used to see fewer ticks than I do now
- No, the number of ticks I see hasn't changed
- Not sure

Q48 How common do you think tick-borne disease is **in humans** in Ohio?

- Very common
- Occurs occasionally
- Rare

Q49 How common do you think tick-borne disease is **in livestock** in Ohio?

- Very common
- Occurs occasionally
- Rare

Q50 Do you think that your work on the farm puts you at higher risk for tick-borne disease than the average person in Ohio?

- Yes
- No

End of Block: Attitudes

Start of Block: Tick Exposure & Prevention Practices: Humans

Q51 Approximately how many ticks have you found on yourself in the past year?

- None
- 1-5
- 6-10
- More than 10

Q52 Where do you encounter ticks most frequently?

- At home
- At work
- During leisure activities (not at home or work)
- Other: __________________________________________________
- I don't encounter ticks

Q53 Has your doctor ever discussed tick-borne disease with you?

- Yes
- No
- I don't have a primary care doctor

Q54 When you go to the doctor, do you tell them about your tick exposure?

- Yes
- No, this is not a topic discussed
- I don't go to the doctor
- I am not exposed to ticks

Q55 Have you ever had a tick-borne disease?

- Yes: My doctor diagnosed me with a specific tick-borne disease
- Maybe: I or my doctor suspected a tick-borne disease and started treatment without a specific diagnosis.
- No: I have not had a tick-borne disease
- Prefer not to answer

Q56 Which tick-borne disease(s) were you diagnosed with? (check all that apply)

- Alpha Gal Syndrome (Red Meat Allergy)
- Anaplasmosis
- Babesiosis
- Ehrlichiosis
- Lyme Disease
- Powassan Virus
- Spotted Fever Rickettsiosis (e.g. Rocky Mountain Spotted Fever)
- Tick Paralysis
- Tularemia
- Other: __________________________________________________
- Not sure

Q57 Which of these personal tick bite prevention strategies do you use when going into areas that you know have a lot of ticks? [check all that apply]

- Wearing long sleeves and long pants
- Using tick repellent (spray, lotion, or repellent-impregnated clothing)
- Checking for ticks soon after leaving the area
- Shower and changing clothes soon after leaving the area
- Other: _________________________________________________
- None

Q58 How often do you use these strategies when you are in areas that you know have a lot of ticks?

- Always
- Often
- Sometimes
- Rarely
- Never

End of Block: Tick Exposure & Prevention Practices: Humans

Start of Block: Tick Exposure & Prevention Practices: Animals

Q60 At the time of year when ticks are most common in your area, how many ticks do you find on a single animal?

- My livestock don't get ticks
- 1-10
- 11-49
- More than 50

Q61 Have you ever seen lots of bugs on your animals that look like tiny spiders or mites?

- Yes
- No

Q62 Have livestock on the farm ever been diagnosed with any of the following diseases?

|  | Yes | No | Not sure |
| --- | --- | --- | --- |
| Anaplasmosis (Gall Sickness) |  |  |  |
| Anemia (specifically associated with tick infestation) |  |  |  |
| Bovine Theileriosis |  |  |  |
| Gotch Ear |  |  |  |
| Lyme Disease (*Borrelia burgdorferi*) |  |  |  |
| Q-Fever (*Coxiella burnetii*) |  |  |  |
| Tularemia |  |  |  |
| Other tick-borne disease: |  |  |  |

Q63 Has the farm's veterinarian discussed tick prevention strategies with animal caretakers on the farm?

- Yes, in the past year
- Yes, but not in the past year
- No
- Not sure

Q64 Which of the following methods do you use to protect livestock on the farm from ticks? (Check all that apply)

- Physical removal
- Spray
- Dust
- Pour-on
- Dip
- Oral/injectable dewormer
- Insecticidal ear tag
- Environmental management (eg. clearing brush)
- Other:
- We don't use any tick prevention methods

Q65 How do you remove ticks from yourself or your animals? [Check all that apply]

- Grasp the tick close to its head/mouth using tweezers and pull straight out
- Grasp the tick close to its head/mouth and remove using a twisting motion
- "Smother" the tick with nail polish, petroleum jelly, alcohol, gasoline, or other substances
- Burn the tick with a match or lighter
- Freeze the tick
- Crush the tick before removing
- Other: __________________________________________________
- I have never removed a tick

Q66 Have you ever submitted a tick for identification?

- Yes
- No

Q67 Why haven't you submitted a tick for identification?

- Did not know this service was available
- Too expensive
- Too inconvenient
- Not concerned about identifying ticks

Q68 Which of the following options would make you more likely to submit ticks for identification? [Check all that apply]

- Submitting photos
- Mailing in the tick
- Submitting the tick through a drop box in your community
- Other: _______________________________________________
- I probably wouldn't submit ticks regardless of what options were available

End of Block: Tick Exposure & Prevention Practices: Animals

Start of Block: Demographics

Q1 What is your age?

________________________________________________________________

Q2 How would you describe yourself?

- Man
- Woman
- Non-binary
- Prefer to self-describe: __________________________________________________
- Prefer not to say

Q3 How would you describe yourself?

- Asian or Pacific Islander
- Black or African American
- Hispanic or Latino
- Native American or Alaskan Native
- White or Caucasian
- Multiracial or Biracial
- A race/ethnicity not listed here

Q4 What is your preferred language?

- English
- Spanish
- Other: __________________________________________________

Q5 What is the highest level of education you have completed? (If you are currently enrolled in school, please select the highest level of education you have completed to date)

- Less than a high school diploma
- High school diploma or equivalent (e.g. GED)
- Technical, trade, or some college
- Associates degree (e.g. AA, AS)
- Bachelor's degree (e.g. BA, BS)
- Master's degree (e.g. MA, MS, MBA)
- Professional Degree or Doctorate (e.g. MD, DVM, PhD)

Q13 What is your annual household income?

- Less than $55,000
- $55,000-$130,000
- More than $130,000

Q14 What county do you live in?

Adams Allen Ashland Ashtabula Athens Auglaize Belmont Brown Butler Carroll Champaign Clark Clermont Clinton Columbiana Coshocton Crawford Cuyahoga Darke Defiance Delaware Erie Fairfield Fayette Franklin Fulton Gallia Geauga Greene Guernsey Hamilton Hancock Hardin Harrison Henry Highland Hocking Holmes Huron Jackson Jefferson Knox Lake Lawrence Licking Logan Lorain Lucas Madison Mahoning Marion Medina Meigs Mercer Miami Monroe Montgomery Morgan Morrow Muskingum Noble Ottawa Paulding Perry Pickaway Pike Portage Preble Putnam Richland Ross Sandusky Scioto Seneca Shelby Stark Summit Trumbull Tuscarawas Union Van Wert Vinton Warren Washington Wayne Williams Wood Wyandot

Q15 Do you live on the farm?

- Yes
- No

Q16 Which of the following describes your role on the farm?

- Farm owner
- Farm employee
- Other: __________________________________________________

Q17 Is your work on the farm your sole employment?

- Yes, I work full-time (40+ hours) on the farm
- Yes, I work part-time (<40 hours) on the farm
- No, I also have another job. Please describe: __________________________________________________

Q18 How many years of experience do you have working on a farm?

- <5 years
- 5-15 years
- >15 years

End of Block: Demographics
