# Supplementary material for: Describing the experience of livestock producers from Ohio, USA with ticks and associated diseases
Source: One Health Outlook. 2023 Nov 20;5:15. doi: 10.1186/s42522-023-00091-4 (PMC10662443; doi:10.1186/s42522-023-00091-4)
Supplement: Supplementary file 2 — Additional file 2: Fig. 1. Number of livestock producers (indicated by color) from each county in Ohio that participated in an electronic survey regarding ticks and tick-borne diseases. [file 42522_2023_91_MOESM2_ESM.docx]

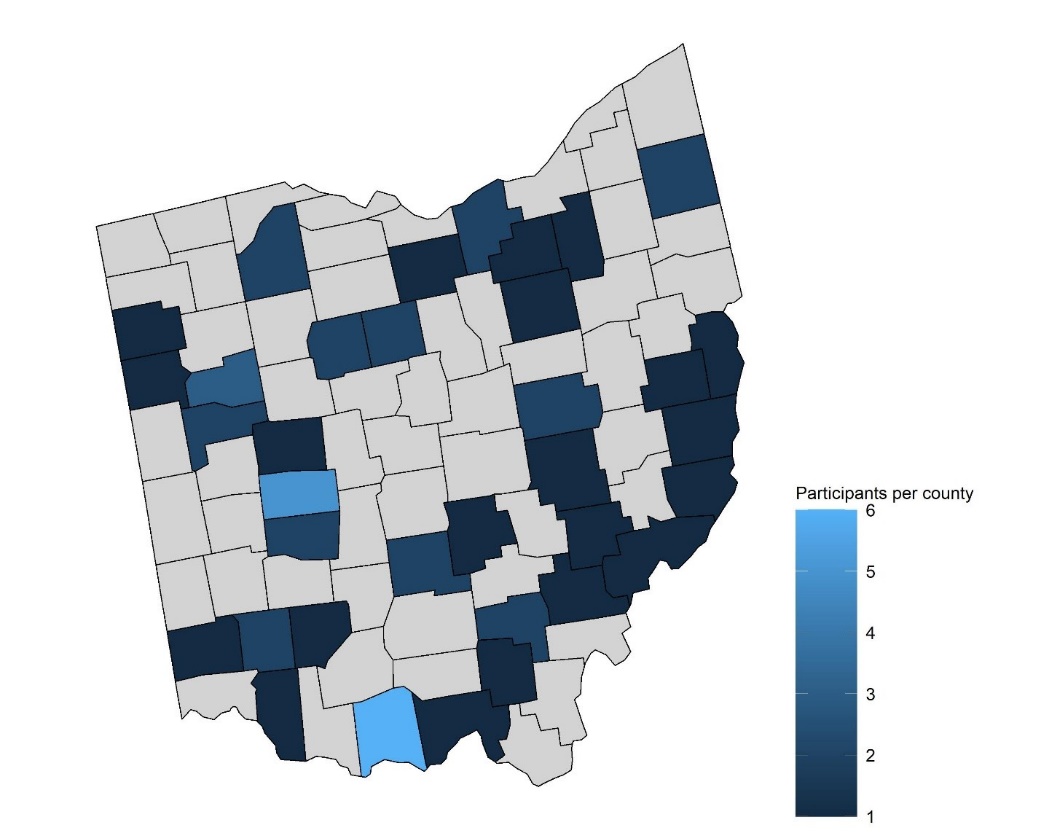


Additional file 2. Fig. 1. Number of livestock producers (indicated by color) from each county in Ohio that participated in an electronic survey regarding ticks and tick-borne diseases.
