# Supplementary material for: Describing the experience of livestock producers from Ohio, USA with ticks and associated diseases
Source: One Health Outlook. 2023 Nov 20;5:15. doi: 10.1186/s42522-023-00091-4 (PMC10662443; doi:10.1186/s42522-023-00091-4)
Supplement: Supplementary file 3 — Additional file 3: Fig. 2. Cluster analysis of responses from livestock producers (n = 48) that participated in an electronic survey regarding ticks and tick-borne diseases (TBDs). A. Factor map demonstrates three clusters (outlined by color) with their respective centers made up of all participants. B. Dendrogram demonstrates the breakdown of participants according to clusters (outlined by color). Variables used for clustering included attitudes towards TBD risk to human or animal health (three categories: very common, occurs occasionally, rare), attitudes towards tick risk to the health of humans (two categories: major issues, minor issues) or animals (three categories: major issues, minor issues, none), and frequency of preventative measures for humans (three categories: always, often, sometimes). The number of preventative measures used in animals (two categories: zero or one, two or more) was deemed insignificant. More details in Additional file 8: Table 5. [file 42522_2023_91_MOESM3_ESM.docx]

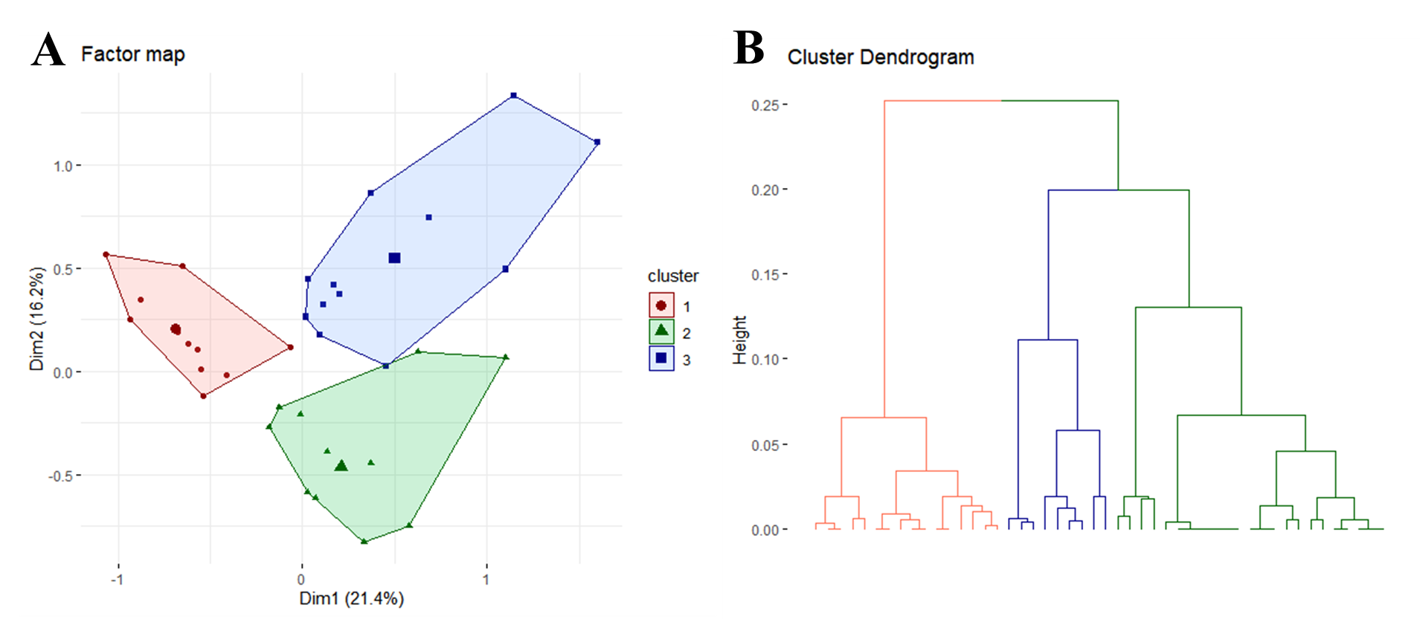


Additional file 3. Fig. 2. Cluster analysis of responses from livestock producers (*n* = 48) that participated in an electronic survey regarding ticks and tick-borne diseases (TBDs). A. Factor map demonstrates three clusters (outlined by color) with their respective centers made up of all participants. B. Dendrogram demonstrates the breakdown of participants according to clusters (outlined by color). Variables used for clustering included attitudes towards TBD risk to human or animal health (three categories: very common, occurs occasionally, rare), attitudes towards tick risk to the health of humans (two categories: major issues, minor issues) or animals (three categories: major issues, minor issues, none), and frequency of preventative measures for humans (three categories: always, often, sometimes). The number of preventative measures used in animals (two categories: zero or one, two or more) was deemed insignificant. More details in Additional Table 5.
