# Supplementary material for: Describing the experience of livestock producers from Ohio, USA with ticks and associated diseases
Source: One Health Outlook. 2023 Nov 20;5:15. doi: 10.1186/s42522-023-00091-4 (PMC10662443; doi:10.1186/s42522-023-00091-4)
Supplement: Supplementary file 4 — Additional file 4: Table 1. Description of the livestock species raised on the farms of Ohio-based producers (n = 57) that participated in an electronic survey regarding ticks and tick-borne diseases. Number of responses (with percentage) are shown for each question. [file 42522_2023_91_MOESM4_ESM.docx]

Additional file 4: Table 1. Description of the livestock species raised on the farms of Ohio-based producers (*n* = 57) that participated in an electronic survey regarding ticks and tick-borne diseases. Number of responses (with percentage) are shown for each question.

| Name of Species | Is this species present on the farm? | | If yes, how many weaned animals are present on the farm? | |  |
| --- | --- | --- | --- | --- | --- |
| Camelids (e.g. llamas, alpacas) | No   Yes  No answer | 19 (33.3%)  29 (50.9%)  9 (15.8%) | Fewer than 10  10 to 50  More than 50  No answer | 5 (8.8%)  21 (36.8%)  3 (5.3%)  0 (0%) | |
| Cattle, beef | No   Yes  No answer | 16 (28.1%)  36 (63.2%)  5 (8.8%) | Fewer than 20  20 to 100  More than 100  No answer | 13 (22.8%)  20 (35.1%)  2 (3.5%)  1 (1.7%) | |
| Cattle, dairy | No  Yes  No answer | 17 (28.8%)  31 (54.4%)  9 (15.8%) | Fewer than 20  20 to 200  More than 200  No answer | 6 (10.5%)  20 (35.1%)  5 (8.8%)  0 (0%) | |
| Cervids (e.g. deer, elk) or bison | No  Yes  No answer | 20 (35.1%)  28 (49.1%)  9 (15.8%) | Fewer than 10  10 to 50  More than 50  No answer | 11 (19.3%)  15 (26.3%)  2 (3.5%)  0 (0%) | |
| Equids (e.g. horses, donkeys) | No  Yes  No answer | 20 (35.1%)  28 (49.1%)  9 (15.8%) | Fewer than 25  25 to 50  More than 50  No answer | 12 (21.1%)  10 (17.5%)  5 (8.8%)  1 (1.7%) | |
| Poultry (e.g. chickens, turkeys, ducks) | No  Yes  No answer | 17 (29.8%)  33 (57.9%)  7 (12.3%) | Fewer than 20,000  20,000 to 100,000  More than 100,000  No answer | 20 (35.1%)  9 (15.8%)  4 (7.0%)  0 (0%) | |
| Small ruminants (e.g. sheep, goats) | No  Yes  No answer | 18 (31.6%)  32 (56.1%)  7 (12.3%) | Fewer than 25  25 to 100  More than 100  No answer | 9 (15.8%)  18 (31.6%)  5 (8.8%)  0 (0%) | |
| Swine | No  Yes  No answer | 31 (54.4%)  16 (28.1%)  10 (17.5%) | Fewer than 25  25 to 1000  More than 1000  No answer | 6 (10.5%)  8 (14.0%)  2 (3.5%)  0 (0%) | |
| Rabbits | No  Yes  No answer | 21 (36.8%)  27 (47.4%)  9 (15.8%) | Fewer than 100  100 to 600  More than 600  No answer | 13 (22.8%)  10 (17.5%)  4 (7.0%)  0 (0%) | |
