# Supplementary material for: Describing the experience of livestock producers from Ohio, USA with ticks and associated diseases
Source: One Health Outlook. 2023 Nov 20;5:15. doi: 10.1186/s42522-023-00091-4 (PMC10662443; doi:10.1186/s42522-023-00091-4)
Supplement: Supplementary file 5 — Additional file 5: Table 2. Knowledge regarding Ohio-relevant tick species of Ohio-based producers (n = 57) that participated in an electronic survey regarding ticks and tick-borne diseases (pictures for each tick were included). Number of responses (with percentage) are shown for each question. [file 42522_2023_91_MOESM5_ESM.docx]

Additional file 5: Table 2. Knowledge regarding tick species of Ohio-based producers (*n* = 57) that participated in an electronic survey (pictures for each tick were included). Number of responses (with percentage) are shown for each question.

| Tick Species | Have you heard of this species? | | Do you think this species is present in Ohio? | | |
| --- | --- | --- | --- | --- | --- |
| American dog tick (*Dermacentor variabilis*) | No   Yes  No answer | 4 (7.0%)  49 (86.0%)  4 (7.0%) | No   Yes  Not sure  No answer | 8 (14.0%)        30 (52.6%)        17 (29.8%)        2 (3.5%) | |
| Asian longhorned tick  (*Haemaphysalis longicornis*) | No   Yes  No answer | 18 (31.6%)  36 (63.2%)  3 (5.3%) | No  Yes  Not sure  No answer | 13 (22.8%)        16 (28.1%)        25 (43.9%)        3 (5.3%) | |
| Blacklegged/Deer tick  (*Ixodes scapularis*) | No  Yes  No answer | 14 (24.6%)  41 (71.9%)  2 (3.5%) | No  Yes  Not sure  No answer | | 12 (21.0%)  21 (36.8%)  21 (36.8%)  3 (5.3%) |
| Brown dog tick  (*Rhipicephalus sanguineus*) | No  Yes  No answer | 21 (36.8%)  33 (57.9%)  3 (5.3%) | No  Yes  Not sure  No answer | | 12 (21.0%)  21 (36.8%)  22 (38.6%)  2 (3.5%) |
| Ear tick  (*Otobius* spp.) | No  Yes  No answer | 23 (40.3%)  31 (54.4%)  3 (5.3%) | No  Yes  Not sure  No answer | | 7 (12.3%)  14 (24.6%)  33 (57.9%)  3 (5.3%) |
| Gulf coast tick  (*Amblyomma maculatum*) | No  Yes  No answer | 26 (45.6%)  28 (49.1%)  3 (5.3%) | No  Yes  Not sure  No answer | | 15 (26.3%)  6 (10.5%)  32 (56.1%)  7.0% (4/57) |
| Lonestar tick  (*Amblyomma americanum*) | No  Yes  No answer | 18 (31.6%)  36 (63.2%)  3 (5.3%) | No  Yes  Not sure  No answer | | 12 (21.0%)  9 (15.8%)  28 (49.1%)  8 (14.0%) |
