# Supplementary material for: Describing the experience of livestock producers from Ohio, USA with ticks and associated diseases
Source: One Health Outlook. 2023 Nov 20;5:15. doi: 10.1186/s42522-023-00091-4 (PMC10662443; doi:10.1186/s42522-023-00091-4)
Supplement: Supplementary file 6 — Additional file 6: Table 3. Knowledge regarding human and animal tick-borne diseases of Ohio-based producers (n = 57) that participated in an electronic survey regarding ticks and tick-borne diseases. Number of responses (with percentage) are shown for each question. [file 42522_2023_91_MOESM6_ESM.docx]

Additional file 6: Table 3. Knowledge regarding human and animal tick-borne diseases of Ohio-based producers (*n* = 57) that participated in an electronic survey. Number of responses (with percentage) are shown for each question.

| Name of Disease | Have you heard of this disease? | | Do you think it occurs in Ohio? | |
| --- | --- | --- | --- | --- |
| African swine fever | No   Yes  No answer | 6 (10.5%)  39 (68.4%)  12 (21.1%) | No   Yes  Not sure  No answer | 11 (19.3%)  12 (21.1%)  21 (36.8%)  13 (22.8%) |
| Alpha gal syndrome (aka Red Meat Allergy) | No   Yes  No answer | 25 (43.9%)  20 (35.0%)  12 (21.1%) | No  Yes  Not sure  No answer | 14 (24.5%)  12 (21.1%)  17 (29.9%)  14 (24.5%) |
| Human anaplasmosis | No  Yes  No answer | 15 (26.3%)  30 (52.6%)  12 (21.1%) | No  Yes  Not sure  No answer | 11 (19.3%)  13 (22.8%)  20 (35.1%)  13 (22.8%) |
| Animal anaplasmosis | No  Yes  No answer | 12 (21.1%)  33 (57.8%)  12 (21.1%) | No  Yes  Not sure  No answer | 10 (17.5%)  12 (21.1%)  22 (38.5%)  13 (22.8%) |
| Human babesiosis | No  Yes  No answer | 21 (36.8%)  24 (42.1%)  12 (21.1%) | No  Yes  Not sure  No answer | 14 (24.6%)  6 (10.5%)  22 (38.6%)  15 (26.3%) |
| Animal babesiosis (aka piroplasmosis, red water, tick fever) | No  Yes  No answer | 23 (40.4%)  22 (38.5%)  12 (21.1%) | No  Yes  Not sure  No answer | 13 (22.8%)  8 (14.0%)  23 (40.4%)  13 (22.8%) |
| Borreliosis (aka Lyme disease) | No  Yes  No answer | 11 (19.3%)  34 (59.6%)  12 (21.1%) | No  Yes  Not sure  No answer | 11 (19.3%)  18 (31.5%)  14 (24.6%)  14 (24.6%) |
| Bovine theileriosis | No  Yes  No answer | 25 (43.9%)  19 (33.3%)  13 (22.8%) | No  Yes  Not sure  No answer | 14 (24.6%)  10 (17.5%)  20 (35.1%)  13 (22.8%) |
| Human ehrlichiosis | No  Yes  No answer | 23 (40.4%)  21 (36.8%)  13 (22.8%) | No  Yes  Not sure  No answer | 12 (21.1%)  9 (15.9%)  22 (38.5%)  14 (24.5%) |
| Animal ehrlichiosis (aka heartwater) | No  Yes  No answer | 22 (38.5%)  23 (40.4%)  12 (21.1%) | No  Yes  Not sure  No answer | 15 (26.3%)  11 (19.3%)  18 (31.6%)  13 (22.8%) |
| Powassan virus | No  Yes  No answer | 25 (43.8%)  20 (35.1%)  12 (21.1%) | No  Yes  Not sure  No answer | 17 (21.0%)  6 (10.5%)  21 (36.7%)  13 (22.8%) |
| Q fever (*Coxiella burnetii*) | No  Yes  No answer | 23 (40.4%)  21 (36.8%)  13 (22.8%) | No  Yes  Not sure  No answer | 16 (28.1%)  6 (10.5%)  21 (36.8%)  14 (24.6%) |
| Spotted Fever Rickettsiosis (e.g., Rocky Mountain Spotted Fever) | No  Yes  No answer | 14 (24.5%)  31 (54.4%)  12 (21.1%) | No  Yes  Not sure  No answer | 13 (22.8%)  12 (21.1%)  17 (29.8%)  15 (26.3%) |
| Tick paralysis | No  Yes  No answer | 9 (15.7%)  36 (63.2%)  12 (21.1%) | No  Yes  Not sure  No answer | 7 (12.3%)  20 (35.1%)  16 (28.1%)  14 (24.5%) |
| Tularemia | No  Yes  No answer | 17 (29.8%)  26 (45.6%)  14 (24.6%) | No  Yes  Not sure  No answer | 12 (21.1%)  14 (24.5%)  16 (28.1%)  15 (26.3%) |
