# Supplementary material for: Describing the experience of livestock producers from Ohio, USA with ticks and associated diseases
Source: One Health Outlook. 2023 Nov 20;5:15. doi: 10.1186/s42522-023-00091-4 (PMC10662443; doi:10.1186/s42522-023-00091-4)
Supplement: Supplementary file 7 — Additional file 7: Table 4. Livestock exposures to tick-borne diseases (TBDs) reported by Ohio-based producers (n = 57) that participated in an anonymous online survey regarding ticks and TBDs. [file 42522_2023_91_MOESM7_ESM.docx]

Additional file 7: Table 4. Livestock exposures to tick-borne diseases (TBDs) reported by Ohio-based producers (*n* = 57) that participated in an anonymous online survey regarding ticks and TBDs.

| Name of Disease | Have livestock on the farm ever been diagnosed with any of the following diseases? | |
| --- | --- | --- |
| Anaplasmosis (Gall Sickness) | No   Yes  Not sure  No answer | 29 (50.8%)  15 (26.3%)  6 (10.5%)  7 (12.3%) |
| Anemia (specifically associated with tick infestation) | No   Yes  Not sure  No answer | 23 (40.4%)  19 (33.3%)  8 (14.0%)  7 (12.3%) |
| Bovine Theileriosis | No  Yes  Not sure  No answer | 27 (47.4%)  13 (22.8%)  9 (15.8%)  8 (14.0%) |
| Gotch Ear | No  Yes  Not sure  No answer | 22 (38.6%)  18 (31.6%)  9 (15.8%)  8 (14.0%) |
| Lyme disease (*Borrelia burgdorferi*) | No  Yes  Not sure  No answer | 26 (45.6%)  11 (19.3%)  12 (21.1%)  8 (14.0%) |
| Q fever (*Coxiella burnetii*) | No  Yes  Not sure  No answer | 33 (57.9%)  5 (8.8%)  11 (19.3%)  8 (14.0%) |
| Tularemia | No  Yes  Not sure  No answer | 22 (38.6%)  16 (28.1%)  11 (19.3%)  8 (14.0%) |
