# Supplementary material for: Describing the experience of livestock producers from Ohio, USA with ticks and associated diseases
Source: One Health Outlook. 2023 Nov 20;5:15. doi: 10.1186/s42522-023-00091-4 (PMC10662443; doi:10.1186/s42522-023-00091-4)
Supplement: Supplementary file 8 — Additional file 8: Table 5. Results from a cluster analysis of responses from Ohio-based livestock producers (n = 48) that participated in an electronic survey regarding ticks and tick-borne diseases (TBDs) in Ohio. Variables used for clustering included attitudes towards TBD risk to human or animal health (three categories: very common, occurs occasionally, rare), attitudes towards tick risk to the health of humans (two categories: major issues, minor issues) or animals (three categories: major issues, minor issues, none), frequency of preventative measures for humans (three categories: always, often, sometimes) and number of preventative measures used in animals (two categories: zero or one, two or more). Variable v-test statistics and p-values are shown for each cluster that was identified. [file 42522_2023_91_MOESM8_ESM.docx]

Additional file 8: Table 5. Results from a cluster analysis of responses from Ohio-based livestock producers (*n* = 48) that participated in an electronic survey regarding ticks and tick-borne diseases in Ohio. Variables used for clustering included attitudes towards TBD risk to human or animal health (three categories: very common, occurs occasionally, rare), attitudes towards tick risk to the health of humans (two categories: major issues, minor issues) or animals (three categories: major issues, minor issues, none), frequency of preventative measures for humans (three categories: always, often, sometimes) and number of preventative measures used in animals (two categories: zero or one, two or more). Variable v-test statistics and p-values are shown for each cluster that was identified.

| Cluster | Variable | v-test statistic | P-value |
| --- | --- | --- | --- |
| 1 | TBD animal risk – Very common | 4.718745 | 2.373042e-06 |
| 1 | TBD human risk – Very common | 4.586533 | 4.506665e-06 |
| 1 | Tick animal risk – Yes, major health risks | 3.351970 | 8.023858e-04 |
| 1 | Prevention frequency – Always | 2.432275 | 1.500430e-02 |
| 1 | Tick human risk – Yes, major health risks | 2.357426 | 1.840214e-02 |
| 1 | TBD human risk – Rare | -2.088043 | 3.679394e-02 |
| 1 | Prevention frequency – Sometimes | -2.273498 | 2.299621e-02 |
| 1 | Tick human risk – Yes, minor health risks | -2.357426 | 1.840214e-02 |
| 1 | TBD human risk – Occurs occasionally | -2.465659 | 1.367616e-02 |
| 1 | Tick animal risk – Yes, minor health risks | -2.654847 | 7.934448e-03 |
| 1 | TBD animal risk – Occurs occasionally | -3.972324 | 7.117486e-05 |
| 2 | Prevention frequency – Often | 4.678353 | 2.891883e-06 |
| 2 | TBD human risk – Occurs occasionally | 4.442011 | 8.912219e-06 |
| 2 | Tick animal risk – Yes, minor health risks | 2.983129 | 2.853179e-03 |
| 2 | TBD animal risk – Occurs occasionally | 2.386334 | 1.701728e-02 |
| 2 | Tick animal risk – Yes, major health risks | 2.420966 | 1.547932e-02 |
| 2 | Prevention frequency – Always | -2.612254 | 8.994732e-03 |
| 2 | Prevention frequency – Sometimes | -2.989473 | 2.794590e-03 |
| 2 | TBD human risk – Very common | -3.442192 | 5.770206e-04 |
| 2 | TBD animal risk – Very common | -3.529025 | 4.170938e-04 |
| 3 | Prevention frequency – Sometimes | 5.277192 | 1.311783e-07 |
| 3 | TBD human risk – Occurs rarely | 3.949905 | 7.818227e-05 |
| 3 | Tick animal risk – No | 1.997315 | 4.579093e-02 |
| 3 | TBD human risk – Occurs occasionally | -2.090218 | 3.659819e-02 |
| 3 | Prevention frequency – Often | -4.773765 | 1.808133e-06 |
