# Supplementary material for: Describing the experience of livestock producers from Ohio, USA with ticks and associated diseases
Source: One Health Outlook. 2023 Nov 20;5:15. doi: 10.1186/s42522-023-00091-4 (PMC10662443; doi:10.1186/s42522-023-00091-4)
Supplement: Supplementary file 9 — Additional file 9: Table 6. Description of demographic and farm-related variables for Ohio-based livestock producers (n = 48) according to their assigned cluster. Number of participants in each cluster are shown and count percentages are shown for each variable. For more details on clusters see the main text and Additional file 8: Table 5. [file 42522_2023_91_MOESM9_ESM.docx]

Additional file 9: Table 6. Description of demographic and farm-related variables for Ohio-based livestock producers (*n* = 48) according to their assigned cluster. Number of participants in each cluster are shown and frequency percentages are shown for each variable. For more details on clusters see the main text and Additional file 8: Table 5.

| Cluster | Median  age (range) | Highest education^b^ | | Lives on farm | | Role  on farm^c^ | | Sole employment^d^ | | Experience (years) | | Purpose of farm | | Median acres cultivated (range) | Most common land type |
| --- | --- | --- | --- | --- | --- | --- | --- | --- | --- | --- | --- | --- | --- | --- | --- |
| 1 (*n* =15)  Aware yet incautious | 37  (27-55) | HS  >HS | 6.7  93.3 | Yes  No | 73.3  26.7 | O  E | 46.7  53.3 | FT  PT  N | 73.3  26.7  0 | <5  5-15  >15 | 40.0  53.3  6.7 | Household  Commercial  Hobby | 0  66.7  33.3 | 50^e^  (5-2000) | Lawn or short pasture |
| 2 (*n* =21)  Aware & cautious | 41^a^  (25-64) | HS  >HS  NA | 28.6  66.6  4.8 | Yes  No  NA | 71.4  19.1  9.5 | O  E  NA | 76.2  19.0  4.8 | FT  PT  N  NA | 52.4  23.8  19.0  4.8 | <5  5-15  >15  NA | 19.0  47.6  28.6  4.8 | Household  Commercial  Hobby | 4.8  61.9  33.3 | 109^f^  (20-5000) | Row crops &  prairie/field |
| 3 (*n* =12)  Unaware & incautious | 42  (28-52) | HS  >HS | 25.0  75.0 | Yes  No | 58.0  42.0 | O  E | 33.3  66.7 | FT  PT  NA | 41.7  33.3  25.0 | <5  5-15  >15 | 33.3  50.0  16.7 | Household  Commercial  Hobby | 16.7  75.0  8.3 | 67.5  (1-200) | Lawn or short pasture |

^a Calculated on 18 responses^

^b HS = High school, NA = no answer^

^c O = Owner, E= Employee, NA = no answer^

^d FT = Yes, full-time, PT= Yes, part-time, N=No, has other job^

^e Calculated on 13 responses^

^f Calculated on 17 responses^
