# Supplementary material for: Describing the experience of livestock producers from Ohio, USA with ticks and associated diseases
Source: One Health Outlook. 2023 Nov 20;5:15. doi: 10.1186/s42522-023-00091-4 (PMC10662443; doi:10.1186/s42522-023-00091-4)
Supplement: Supplementary file 10 — Additional file 10: Table 7. Description of knowledge, exposure, surveillance, and education variables for Ohio-based livestock producers (n = 48) according to their assigned cluster. Number of participants in each cluster are shown and count percentages are shown for each variable. For more details on clusters see the main text and Additional file 8: Table 5. [file 42522_2023_91_MOESM10_ESM.docx]

Additional file 10: Table 7. Description of knowledge, exposure, surveillance, and education variables for Ohio-based livestock producers (*n* = 48) according to their assigned cluster. Number of participants in each cluster are shown and frequency percentages are shown for each variable. For more details on clusters see the main text and Additional file 8: Table 5.

| Cluster | Confident identifying a tick^a^ | | Confident identifying a fed tick^a^ | | Highest perceived  tick exposure | | Place of most frequent tick exposure | | Discuss tick exposure at doctor | | Submitted a tick ever | | Discuss tick prevention with veterinarian | |
| --- | --- | --- | --- | --- | --- | --- | --- | --- | --- | --- | --- | --- | --- | --- |
| 1 (*n* =15)  Aware yet incautious | N  S  M  V | 6.6  26.7  66.7  0 | N  S  M  V | 6.7  40.0  40.0  13.3 | Crop-related acts  Hunting  Handling animals  Hiking/outdoors | 33.3  0  60.0  6.7 | Home  Work  Leisure  None  Other | 26.7  66.7  6.7  0  0 | Yes  No  Other | 80.0  13.3  6.7 | Yes  No | 86.7  13.3 | Yes, in past year  Yes, but not in past year  No  NA | 73.3  13.3  6.7  6.7 |
| 2 (*n* =21)  Aware & cautious | N  S  M  V | 4.8  47.6  28.6  19.0 | N  S  M  V  NA^b^ | 0  23.8  33.3  19.0  23.8 | Crop-related acts  Hunting  Handling animals  Hiking/outdoors NA | 14.3  9.5  23.8  23.8  28.6 | Home  Work  Leisure  None  Other | 23.8  52.4  23.8  0  0 | Yes  No  Other | 38.1  47.6  14.3 | Yes  No | 66.7  33.3 | Yes, in past year  Yes, but not in past year  No  NA | 47.6  9.5  28.6  14.3 |
| 3 (*n* =12)  Unaware & incautious | N  S  M  V | 8.3  50.0  33.3  8.4 | N  S  M  V | 8.3  25.0  58.3  8.3 | Crop-related acts  Hunting  Handling animals  Hiking/outdoors | 8.3  8.3  58.3  25.1 | Home  Work  Leisure  None  Other | 16.7  58.3  8.3  8.3  8.3 | Yes  No  Other | 50.0  41.7  8.3 | Yes  No  NA | 66.7  25.0  8.3 | Yes, in past year  Yes, but not in past year  No  NA | 33.3  33.3  16.7  16.7 |

^a N= not at all, S = slightly, M=moderately, V=very^

^b NA = no answer^
