# Supplementary material for: Describing the experience of livestock producers from Ohio, USA with ticks and associated diseases
Source: One Health Outlook. 2023 Nov 20;5:15. doi: 10.1186/s42522-023-00091-4 (PMC10662443; doi:10.1186/s42522-023-00091-4)
Supplement: Supplementary file 11 — Additional file 11. R code for cluster analysis. [file 42522_2023_91_MOESM11_ESM.docx]

R code for cluster analysis

#original_data =>raw data with columns for all questions where each row is a producer

library(factoextra) #load required library

library(FactoMineR) # load required library

library(dplyr) #load required library

producer_data=as.data.frame(cbind(original_data$Q42, original_data$Q43, original_data$Q48, original_data$Q49, original_data$Q58, original_data$Q64, original_data$Response_ID) #subset data

producer_data_revised=as.data.frame(lapply(producer_data,factor)) # convert categorical variables to factors

producer_data_revised=select(producer_data_revised,c(-7)) #remove response ID (producer ID)

MCAproducers=MCA(producerdata) # to run multiple correspondence analysis (MCA)

HCPCproducers=HCPC(MCAproducers, metric="Euclidean", method="ward", proba=0.05) #to run hierarchical clustering on principal components and create dendrogram (tree)

HCPCproducers$desc.var$category # to look at each cluster

fviz_cluster(HCPCproducers, geom="point", main="Factor map", ggtheme=theme_minimal()) + scale_colour_manual (values=c(“darkred”,“darkgreen”,”darkblue)) #to create factor map

fviz_dend(HCPCproducers,show_labels=FALSE)+scale_colour_manual(values=c(“blue3”,”green3”,”coral1”,”darkgreen”) # to create tree without producer IDs to match with factor map

producer_data$cluster=HCPCproducers$data.clust$clust #add assigned cluster to truncated dataset

clusterproducer=merge(original_data,producer_data,by=”Response_ID”) #assign clusters to original data

cluster1=subset(clusterproducer,clusterproducer$cluster==1) #interrogate each cluster
